# Clonidine versus Captopril for Severe Postpartum Hypertension: A Randomized Controlled Trial

**DOI:** 10.1371/journal.pone.0168124

**Published:** 2017-01-26

**Authors:** Carlos Noronha Neto C, Sabina S. B. Maia, Leila Katz, Isabela C. Coutinho, Alex R. Souza, Melania M. Amorim

**Affiliations:** Post Graduate Program on Maternal and Child Health, Instituto de Medicina Integral Prof. Fernando Figueira, Recife, Pernambuco, Brazil; Kurume University School of Medicine, JAPAN

## Abstract

**Background:**

Changes during the puerperium are still unclear, particularly in women with hypertension. The choice of antihypertensives, both to control very high blood pressure episodes and to keep blood pressure stable, also requires further elucidation. Currently, there are no clear data to guide the decision for the choice of postpartum antihypertensives. Captopril plays an important role in the treatment of very high blood pressure episodes and may be used postpartum. Clonidine has been used as an alternative in pregnant or postpartum women with contraindications to captopril, with satisfactory effect. The objective of the present study was to evaluate the effectiveness and safety of clonidine compared to captopril for treating severe postpartum hypertension.

**Methods and findings:**

A randomized, drug-controlled, triple-blind clinical trial evaluating postpartum women receiving captopril or clonidine. Inclusion criteria consisted of: women with hypertensive disorders of pregnancy systolic blood pressure (SBP) ≥180 mmHg and/or diastolic blood pressure (DBP) ≥110 mmHg], requiring magnesium sulfate. Exclusion criteria were: heart disease, smoking, illicit drug use, contraindications to captopril, clonidine or oral medication, and having used captopril/clonidine previously. The primary outcome was the frequency of very high blood pressure episodes while in the obstetric intensive care unit. A total of 90 postpartum women met the study inclusion criteria, with 45 randomized to each group. There were fewer very high blood pressure episodes during hospitalization (2.1 ± 2.1 vs. 3.5 ± 4.7, p = 0.08), greater percentage reduction in SBP (14.0% ± 8.6% vs. 10.8% ± 8.8%, p = 0.08) and fewer women requiring sodium nitroprusside (2.3% vs. 13.3%; RR: 0.17; 95%CI: 0.02–1.39; p = 0.06) in the clonidine group compared to the captopril group; however, these differences were not significant. The groups were similar regarding daily mean SBP or DBP; however, on the third postpartum day, mean SBP was lower in the clonidine compared to the captopril group (151.9 ± 11.8 mmHg vs. 158.1 ± 13.6 mmHg, p = 0.02). Although not statistically significant, adverse reactions were more common in the captopril group (28.8%) compared to the clonidine group (18.6%).

**Conclusion:**

Clonidine and captopril represent safe, effective treatments for severe postpartum hypertension.

**Trial registration:**

clinicaltrials.gov: www.clinicaltrial.gov, NCT01761916.

## Introduction

Pregnancy induces changes that not only permit the fetus to develop adequately, but also prepare the woman’s body for childbirth, breastfeeding and the reestablishment of pre-pregnancy conditions [1]. These alterations may directly or indirectly affect maternal blood pressure. However, changes during the puerperium are still unclear, particularly in women with hypertension [2–4].

The choice of antihypertensives, both to control very high blood pressure episodes and to keep blood pressure stable, also requires further elucidation [4,5]. Currently, there are no clear data to guide the decision for the choice of postpartum antihypertensives. [5,6].

Captopril, angiotensin-converting enzyme (ACE) inhibitors, plays an important role in the treatment of very high blood pressure episodes [7] and may be used postpartum with no effect on breastfeeding [8]. Nevertheless, in cases of drug intolerance or acute renal disease, captopril should be avoided [9–10].

Clonidine has been used as an alternative in pregnant or postpartum women with contraindications to captopril, with satisfactory effects [11]. It is a centrally acting alpha-2 agonist with a systemic antihypertensive effect that reduces the tonus of the sympathetic nervous system, promoting hemodynamic stability. Clonidine also has a sedative and anxiolytic effect and reduces the concentration of circulating catecholamines [12].

The objective of the present study was to determine the effectiveness of clonidine compared to captopril for treating severe postpartum hypertension. There are no clinical trials evaluating these aspects in this specific group of postpartum patients.

## Materials and Methods

A randomized, triple-blind, drug-controlled clinical trial was conducted to compare oral captopril (25 mg) with oral clonidine (0.1 mg) for postpartum women with hypertensive disorders of pregnancy and very high blood pressure episodes. The study was developed at the obstetric intensive care unit (ICU) of the Instituto de Medicina Integral Prof. Fernando Figueira (IMIP) in Recife, Pernambuco, northeastern Brazil between November 2012 and June 2013. The study was approved by the institute’s internal review board under reference number 05598812.1.0000.5201 the protocol was registered at ClinicalTrials.gov (www.clinicltrials.gov), reference NCT01761916 and published in Reproductive Health [13]. All the women voluntarily agreed to participate and signed an informed consent form.

The inclusion criteria were: postpartum women with a diagnosis of hypertensive disorders of pregnancy, with very high blood pressure episodes and requiring magnesium sulfate to prevent or treat eclampsia. Women with heart conditions, smokers, users of illicit drugs that could interfere with maternal hemodynamics, those with contraindications to the use of captopril (acute or chronic renal disease, chronic liver disease and hypersensitivity to the drug), contraindications to clonidine (sinus node disease, chronic liver disease and hypersensitivity to the drug), women unable to take oral medication and those who had used captopril or clonidine prior to admission were excluded from the study.

The National High Blood Pressure Education Program (2000) criteria were used to diagnose severe preeclampsia, superimposed preeclampsia and eclampsia [14,15]. A very high blood pressure episode was defined as systolic blood pressure (SBP) ≥180 mmHg and/or diastolic blood pressure (DBP) ≥110 mmHg [16].

All the patients included in the study were identified and admitted to IMIP’s obstetric ICU following delivery. All were given magnesium sulfate (MgSO_4_) intravenously to prevent or control eclampsia in accordance with the practice established in this institution (an attack dose of 6 g IV followed by 1–2 g/hour IV for 24 hours) [15].

During use of the anticonvulsant (MgSO_4_), blood pressure was measured every two hours in the first 24 hours and then every six hours (routine practice in this institute for these patients). Following confirmation of the first episode of very high blood pressure by medical and/or nursing team, the woman was then provided with information about the study and its importance. After signing an informed consent form, the woman was included in the study and allocated a reference number corresponding to the chronological order of admission. Randomization was then carried out.

A total of 90 women were randomized according to a list prepared by a statistician using the Random Allocation software program (Isphahan, Iran), version 1.0. In the list, the letters A and B referred to the two groups. The team pharmacist then allocated the letters A and B to captopril or clonidine. The investigators and the statistician were blinded to this information.

Identical boxes were prepared and numbered sequentially from 1 to 90 in accordance with the randomization list. Each box contained 30 tablets of captopril (25 mg) or clonidine (0.1 mg). Neither the investigators nor the women nor the statistician knew which drug was in which box.

Following inclusion to the study, the box corresponding to that particular patient was given to the nursing technician responsible for that hospital bed to administer the oral medication whenever the woman suffered a very high blood pressure episode. After administering the study drug, blood pressure was measured every 20 minutes until it returned to levels before the episode (systolic blood pressure (SBP) <180 mmHg and diastolic blood pressure (DBP) <110 mmHg. This procedure was repeated at each subsequent very high blood pressure episode. Pressure was measured every two hours in the first 24 hours and every six hours thereafter even if the women had no more very high blood pressure episodes.

To ensure that the maximum daily dose of each drug was not exceeded, it was established that each patient would receive a maximum of six doses/day [captopril (150 mg/day) or clonidine (0.6 mg/day)]. If the dose required exceeded the maximum daily dose, then another antihypertensive drug (nifedipine or hydralazine) was selected to treat very high blood pressure episodes [4]. Sodium nitroprusside was used for women who continued to have very high blood pressure episodes even after other hypertensive drugs were used.

The primary outcome was the frequency of very high blood pressure episodes while in the obstetric ICU. At the time of the protocol we intended to evaluate mean arterial blood pressure [13], but after this we understood that the best primary outcome was in fact the number of very blood pressure episodes, so we modified this before initiating data collection.

The secondary outcomes were: daily mean SBP and DBP levels, the number of days with very high blood pressure episodes, number of days until blood pressure was controlled, percentage reduction in SBP, percentage reduction in DBP, number of doses used to control blood pressure, need to associate another hypertensive agent to control hypertension, number of antihypertensives associated, need for sodium nitroprusside, maternal complications associated with very high blood pressure episodes (acute myocardial infarction, cerebral vascular accident, pulmonary edema and eclampsia), maternal complications unrelated to very high blood pressure episodes (HELLP syndrome, disseminated intravascular coagulation, thromboembolism, encephalopathy and renal or liver failure), adverse effects of the use of the antihypertensives, number of days in the obstetric ICU and death.

Sample size was calculated using the OpenEpi software program (Centers for Disease Control and Prevention, GA, USA). A pilot study was conducted with an initial sample of 30 postpartum women with hypertensive disorders of pregnancy, 15 in each group. The mean number of very high blood pressure episodes during hospitalization in IMIP’s obstetric ICU (2.8 ± 2.0 in the clonidine group and 6.2 ± 6.2 in the captopril group) was then used to calculate sample size, for a power of 90% and a 95% confidence level (two sided t-test). It was found that 78 patients would be required to show this difference; however, it was decided to increase this number to 90 to compensate for any losses or differences between the groups.

Statistical analysis was conducted using the Medcalc software program (Ostend, Belgium), version 12.7.0. Student’s t-test was used for continuous variables with normal distribution. The categorical variables were compared using the Pearson chi-square test of association or Fisher’s exact test, as appropriate. Risk ratios (RR) and their respective 95% confidence intervals were calculated.

Repeated measures analysis of variance was used to compare daily mean SBP and DBP levels in both groups in the first four days postpartum. Sphericity was assumed, and p-values were calculated for the time-by-intervention interaction. Analysis was conducted on an intention-to-treat basis. All p-values were two-tailed and a significance level of 5% was adopted throughout the analysis.

## Results

**Fig 1:**
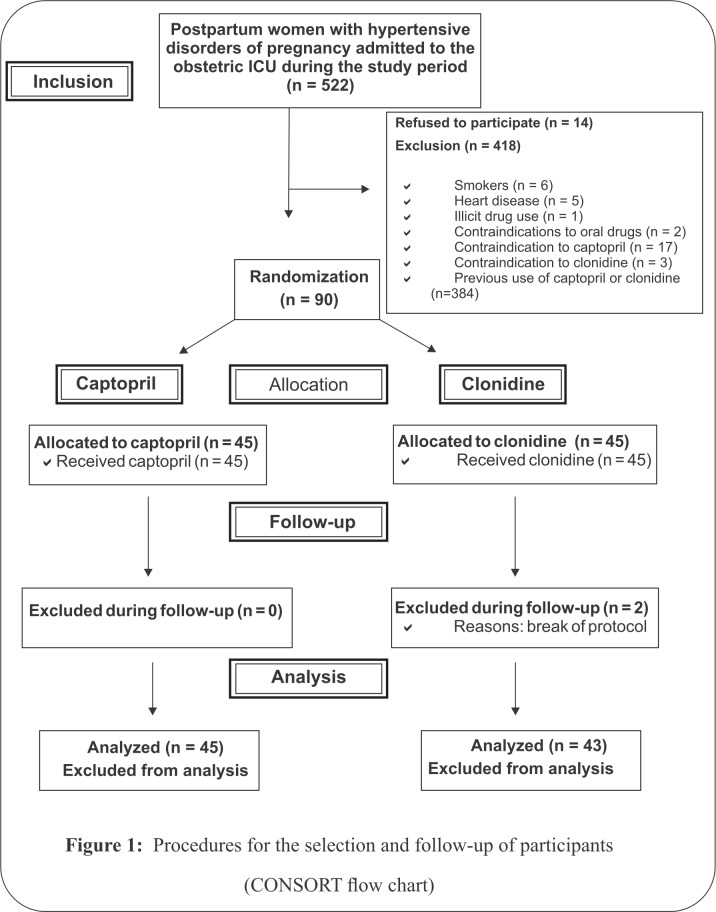
Procedures for the selection and follow-up of participants (CONSORT flow chart).

During the study period, 522 postpartum women with hypertensive disorders of pregnancy were admitted to IMIP’s obstetric ICU. Of these, 14 women declined to participate in the study. Other reasons for exclusion: 384 women had used captopril and/or clonidine previously, six were smokers, five had heart disease, one used illicit drugs, two had contraindications to oral medication, 17 had contraindications to captopril and three had contraindications to clonidine (Fig 1).

A total of 90 postpartum women met the study inclusion criteria, with 45 randomized to each group. There were two exclusions during follow-up, both in the clonidine group after captopril was inadvertently administered by the attending physician. Therefore, 43 women were analyzed in the clonidine group and 45 in the captopril group (Fig 1).

The baseline characteristics of the women in the two groups were similar, with no statistically significant differences. Severe preeclampsia was the most common hypertensive disorder (64.4%) (Table 1).

**Table 1:** Baseline characteristics of the postpartum women with hypertensive disorders of pregnancy.

Characteristic	Clonidine (n = 43)	Captopril (n = 45)
Age (years) mean (SD)	28.9 (6.7)	28.8 (6.7)
Number of pregnancies median (IQR)	2.0 (1.0–3.5)	2.0 (1.0–3.0)
Parity median (IQR)	2.0 (1.0–2.0)	2.0 (1.0–3.5)
Gestational age (weeks) mean (SD)	34.1 (4.0)	35.0 (3.4)
Types of hypertensive disorders		
Severe preeclampsia	27(62.8)	31(68.9)
Imminent eclampsia	4 (9.3)	6 (13.3)
Superimposed preeclampsia	15 (34.8)	9 (20.0)
Eclampsia	3 (6.9)	3 (6.6)
HELLP syndrome	8 (18.6)	11 (24.4)
Blood pressure at admission		
SBP (mmHg) mean (SD)	156.7 (16.7)	161.2 (21.6)
DBP (mmHg) mean (SD)	102.6 (12.0)	102.6 (16.1)
Laboratory parameters		
Platelets (1,000/mm^3^) median (IQR)	185 (57.0–234.5)	158 (53.0–222.5)
Urea (mg/dl) mean (SD)	27.0 (12.3)	26.5 (14.0)
Creatinine (mg/dl) mean (SD)	0.6 (0.2)	0.7 (0.2)
Uric acid (mg/dl) mean (SD)	6.6 (2.6)	6.4 (1.5)
LDH (U/l) mean (SD)	425.4 (281.6)	428.7 (224.1)
AST (U/l) mean (SD)	52.9 (94.4)	54.8 (66.3)
ALT (U/l) mean (SD)	51.7 (100.3)	52.4 (72.3)
TB (mg/dl) mean (SD)	0.2 (0.3)	0.3 (0.3)
DB (mg/dl) mean (SD)	0.1 (0.2)	0.1 (0.1)
IB (mg/dl) mean (SD)	0.1 (0.1)	0.2 (0.2)

SD: standard deviation, IQR: interquartile range, SBP: systolic blood pressure, DBP: diastolic blood pressure, LDH: lactate dehydrogenase, AST: aspartate aminotransferase, ALT: alanine aminotransferase, RR: relative risk, CI: confidence interval, TB: total bilirubin, DB: direct bilirubin, IB: indirect bilirubin.

The frequency of the clinical parameters was also similar in the two groups; however, there were fewer very high blood pressure episodes during hospitalization (2.1 ± 2.1 vs. 3.5 ± 4.7; p = 0.08), a greater percentage reduction in systolic pressure (14.0% ± 8.6% vs. 10.8% ± 8.8%; p = 0.08) and less need for sodium nitroprusside (2.3% vs. 13.3%; RR: 0.17; 95%CI: 0.02–1.39; p = 0.06) in the clonidine group compared to the captopril group (Table 2).

**Table 2:** Clinical parameters following the use of captopril or clonidine for treating very high blood pressure episodes in postpartum women with hypertensive disorders of pregnancy.

Characteristic	Clonidine (n = 43)	Captopril (n = 45)	RR (95%CI)	p-value
Days of hospitalization mean (SD)	4.8(2.0)	4.1(1.9)		0.09^a^
Number of very high blood pressure episodes/day mean (SD)	2.1(2.1)	3.5(4.7)		0.08^a^
Number of days with very high blood pressure episodes mean (SD)	3.3(2.5)	3.0(1.9)		0.52^a^
Number of days until BP control mean (SD)	4.1(2.5)	3.5(2.0)		0.25^a^
% reduction in SBP mean (SD)	14.0(8.6)	10.8(8.8)		0.08^a^
% reduction in DBP mean (SD)	15.6(9.7)	14.9(9.1)		0.73^a^
Number of doses until BP control	2.3(1.9)	2.2(1.6)		0.69^a^
Other antihypertensive drugs n (%)	35(81.4)	37(82.2)	0.98(0.81–1.20)	0.86^b^
Nitroprusside n (%)	1(2.3)	6(13.3)	0.17(0.02–1.39)	0.06^c^

%: percentage, RR: relative risk, CI: confidence interval, SD: standard deviation, IQR: interquartile range, BP: blood pressure, SBP: systolic blood pressure, DBP: diastolic blood pressure.

^a^Student’s t-test.

^b^Pearson’s chi-square test.

^c^Fisher’s exact test.

**Fig 2:**
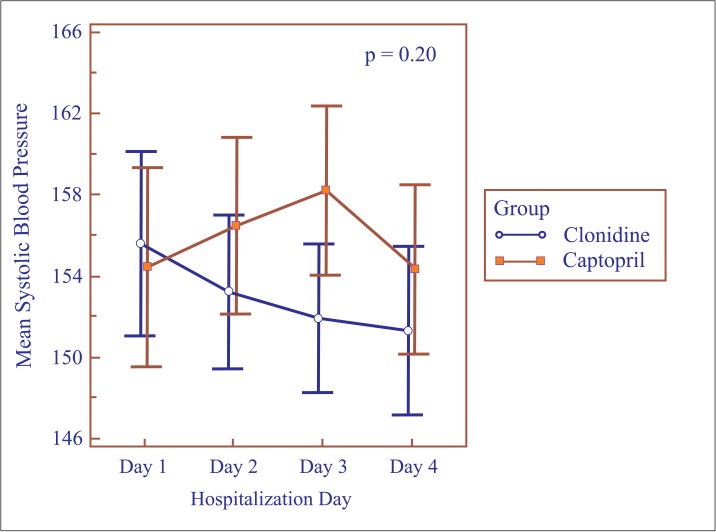
Mean systolic blood pressure according to hospitalization day.

**Fig 3:**
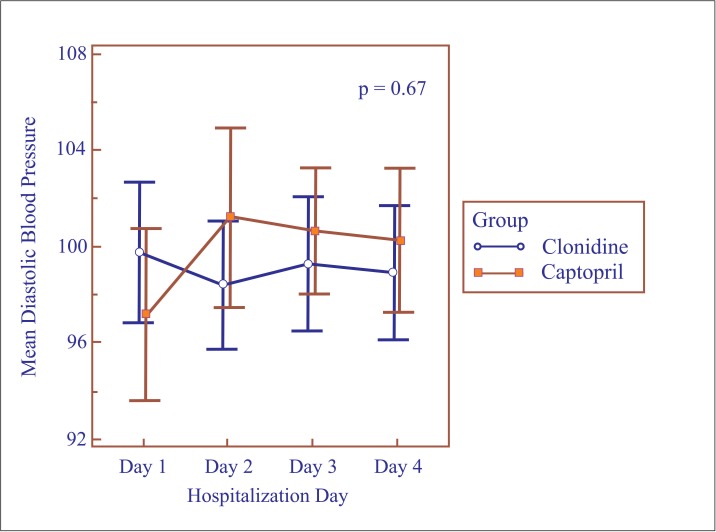
Mean diastolic blood pressure according to hospitalization day.

Repeated measures analysis of variance for the first four days of hospitalization showed no differences between the groups with respect to mean daily SBP (p = 0.20) (Fig 2) or DBP levels (p = 0.67) (Fig 3). Nevertheless, mean SBP was lower on the third day in the clonidine group (151.9 ± 11.8 mmHg vs. 158.1 ± 13.6 mmHg; p = 0.02) (Table 3). The same was not found for DBP, which remained similar in both groups (Table 3).

**Table 3:** Mean blood pressure per hospitalization day in postpartum women with hypertensive disorders of pregnancy.

Characteristic	Clonidine (n = 43)	Captopril (n = 45)	p-value^a^
1^st^ day of hospitalization			
SBP (mmHg) mean (SD)	155.5 (14.6)	154.4 (16.2)	0.73
DBP (mmHg) mean (SD)	99.7 (9.5)	97.1 (11.9)	0.26
2^nd^ day of hospitalization			
SBP (mmHg) mean (SD)	153.2 (12.2)	156.4 (14.4)	0.26
DBP (mmHg) mean (SD)	98.3 (8.6)	101.2 (12.4)	0.22
3^rd^ day of hospitalization			
SBP (mmHg) mean (SD)	151.9 (11.8)	158.1 (13.6)	0.02
DBP (mmHg) mean (SD)	99.3 (9.0)	100.6 (8.6)	0.47
4^th^ day of hospitalization			
SBP (mmHg) mean (SD)	151.3 (13.3)	154.3 (13.8)	0.30
DBP (mmHg) mean (SD)	98.9 (9.1)	100.2 (10.0)	0.52

N: sample size, SD: standard deviation, SBP: systolic blood pressure, DBP: diastolic blood pressure.

^a^Student’s t-test.

During hospitalization, HELLP syndrome was the most frequently diagnosed postpartum complication, both in the clonidine group (30.2%) and in the captopril group (28.8%). Although not statistically significant, adverse reactions (dry cough, rashes, fever, nausea, etc.) were more common in the captopril group (28.8%) compared to the clonidine group (18.6%) (Table 4).

**Table 4:** Postpartum complications and adverse reactions to the use of captopril or clonidine for treating very high blood pressure episodes in postpartum women with hypertensive disorders of pregnancy.

Characteristics	Clonidine (n = 43)	Captopril (n = 45)	RR (95%CI)	p-value
Postpartum complications n (%)	22(44.2)	23 (48.9)	0.90 (0.57–1.41)	0.81^a^
Unassociated with hypertension peak	2(4.6)	3 (6.6)	0.69 (0.12–3.97)	0.52^b^
Associated with peak	5(11.6)	6 (13.3)	0.87 (0.28–2.64)	0.93^a^
Acute pulmonary edema	1(2.3)			
Eclampsia	4(9.3)	2 (4.4)	2.09 (0.40–10.84)	0.31^b^
Imminent eclampsia	3(6.9)	5 (11.1)	0.62 (0.15–2.46)	0.38^b^
Oliguria	4(9.3)	6 (13.3)	0.69 (0.21–2.30)	0.39^b^
HELLP syndrome	13(30.2)	11 (24.4)	1.23 (0.62–2.45)	0.71^a^
Adverse reactions n (%)	8(18.6)	13 (28.8)	0.64 (0.29–1.39)	0.37^a^

n: sample size, %: percentage, RR: relative risk, CI: confidence interval.

^a^Pearson’s chi-square test.

^b^Fisher’s exact test.

None of the women needed to interrupt antihypertensive treatment because of adverse effects and all fulfilled the necessary criteria for discharge from the obstetric ICU. No cases of maternal death were recorded.

## Discussion

No significant differences were found in the clinical parameters of these postpartum women with very high blood pressure episodes. To the best of our knowledge, there are no systematic reviews or clinical trials evaluating these aspects in which oral captopril (25 mg) is compared with oral clonidine (0.1 mg) in this specific group of postpartum patients.

The effects of these drugs alone [17] or in combination with other antihypertensives have been confirmed in groups of non-pregnant patients [18,19]. A double-blind clinical trial compared oral captopril with transdermal clonidine in patients with mild hypertension. After a 2-3-week administration period, SBP/DBP decreased in both groups from 146.3/95.4 to 134.7/85.1 mmHg in 33 patients treated with clonidine and from 143.0/96.1 to 134.8/87.1 mmHg in 35 patients treated with captopril [18].

Although the routes of administration of the antihypertensives were different, the mean number of very high blood pressure episodes was similar to that found in the present study for the clonidine group, but different from that found for the captopril group (2.0 versus 4.9, respectively). This difference may be due to the different route of administration of the clonidine (transdermal). In that clinical trial, four patients in the transdermal clonidine group and one in the oral captopril group were discontinued due to adverse effects [18]. In the present study, no women were discontinued for this reason. There were no reports of sedative effect with both drugs.

As with all angiotensin-converting enzyme (ACE) inhibitors, captopril should not be used in pregnancy [6]. However, both captopril and clonidine can be used postpartum with no effect on lactation [5,6]. In our study breastfeeding was not a concern because all patients were in ICU regimen and not breastfeeding. Anyhow, although some recommend caution with lactation since clonidine is excreted in human milk at concentrations roughly twice that in maternal serum [20] consequences of the use of this drug is rare [21]. Clonidine has been granted a license for the treatment of preeclampsia since the year 2000 [22] and also is considered safe and low risk for use during lactation [23].

Captopril is contraindicated in cases of acute renal failure, a complication that is relatively common in postpartum women with severe preeclampsia or eclampsia [9], and clonidine may constitute an alternative treatment for this particular group of patients. In our service, a frequency of oliguria of 27.9% has been reported [3], as well as a frequency of acute renal disease of 11.7% [9], in hospitalized patients with severe preeclampsia or eclampsia.

Based on the present data, blood pressure control was similar in both groups, suggesting that both drugs are effective for the treatment of very high blood pressure episodes in postpartum women with hypertensive disorders of pregnancy. However, mean SBP was lower in the clonidine group on the third day. In these patients, the reduction in blood pressure levels could possibly be explained by the resolution of the endothelial lesion and vasospasm following delivery, and also by diuresis of the fluid extravasated to the third space, which would be potentiated by the pharmacokinetics of clonidine and its vascular effect [12].

The need for other antihypertensive drugs was high, and similar (around 80%) in both groups. Nevertheless, it is debatable whether or not there is any need to maintain treatment in postpartum women with preeclampsia or eclampsia, and a Cochrane systematic review failed to find any evidence supporting the use of medication to control postpartum hypertension [24].

The frequencies of complications were similar in both groups, with the most common complication being the HELLP syndrome. Although the difference between the two groups regarding the need for other antihypertensive drugs was slight, the need for sodium nitroprusside was greater in the captopril group compared to the clonidine group. There were also fewer very high blood pressure episodes per day in this group, although this difference was not statistically significant. It is possible that with larger sample sizes differences may be identified.

In relation to the cost of the drugs, a 30-tablet package of captopril (25 mg) is sold in Brazil for US$50–80 [25], while a 30-tablet package of clonidine hydrochloride (0.1 mg) costs US$13–20 [26]. Taking the daily frequency of peaks and the mean number of hospitalization days in the obstetric ICU into account, the women used approximately 10 clonidine and 14 captopril tablets, resulting in a mean treatment cost of US$4.20 for the clonidine group and US$23.12 for the captopril group. Therefore, clonidine is more cost-effective than captopril.

This study is important because it investigates another option for the treatment of hypertensive emergency during the postpartum period. It was a well-designed randomized clinical trial and to the best of our knowledge, there are no systematic reviews or clinical trials evaluating these aspects in which oral captopril (25 mg) is compared with oral clonidine (0.1mg) in this specific group of postpartum patients.

The sample size is still small, and maybe a grater sample would be necessary to show more differences in the effect of the two drugs. An analysis of the level of clonidine in breast milk of the patients was also not conducted. It would also have been interesting in conducting a longer observation of the women and evaluating blood pressure levels after discharge from ICU.

External validity is still limited since this was a very selected population of patients, and in a very specific setting and new studies is different hospitals with different populations are still necessary.

## Conclusions

We consider that antihypertensive treatment with clonidine may constitute a safe, effective alternative for avoiding postpartum very high blood pressure episodes, with certain advantages such as a lower treatment costs. To define whether this antihypertensive should be adopted in different services and regions, each hospital should take into consideration the characteristics of the population, the frequency of very high blood pressure episodes in postpartum women and the confidence and ease of the medical team with the use of this drug.

Although no studies of this type have yet been conducted with this particular group of postpartum patients, a systematic review including a metaanalysis of future studies should also be carried out to evaluate outcomes such as the duration of postpartum hospital stay, the occurrence of other complications related to very high blood pressure episodes, the behavior and control of blood pressure postpartum and patient satisfaction.

## Supporting Information

S1 File Study Protocol. DOI: 10.1186/1742-4755-10-37.
(PDF)

S2 File Study Protocol Complete. (PDF)

S3 File CONSORT 2010 checklist. (PDF)

S1 Data ClonidinaCaptopril. (XLSX)

S2 Data Cloncap repeated measures. (XLSX)
