# Supplementary material for: Clonidine versus Captopril for Severe Postpartum Hypertension: A Randomized Controlled Trial
Source: PLoS One. 2017 Jan 26;12(1):e0168124. doi: 10.1371/journal.pone.0168124 (PMC5268489; doi:10.1371/journal.pone.0168124)
Supplement: S2 File — (PDF) [file pone.0168124.s002.pdf]

INSTITUTO DE MEDICINA INTEGRAL PROF. FERNANDO FIGUEIRA

EFETIVIDADE DA CLONIDINA PARA TRATAMENTO HIPOTENSOR DE EMERGÊNCIA NO PUERPÉRIO: ENSAIO CLÍNICO RANDOMIZADO

Projeto de pesquisa do Doutorado em Saúde Materno-Infantil do Instituto de Medicina Integral Prof. Fernando Figueira.

Aluno: Carlos Noronha Neto

Orientadora: Melania Maria Ramos de Amorim

Co-orientadora: Leila Katz

Isabela Cristina Coutinho de Albuquerque Neiva Coêlho

Colaboradore: Sabina Bastos Maia

Junho de 2012

ALUNO: ***Carlos Noronha Neto***
Mestre em Tocoginecologia pela Universidade de Pernambuco (UPE)
Especialista em Tocoginecologia e Medicina Fetal.
Preceptor da Residência Médica em Tocoginecologia e Medicina Fetal do IMIP
Consultor nordeste de violência pelo Ministério da Saúde
RG: 8342384 SDS-PE CPF: 938851995-72
Telefones: (81) 3241-5876 (81) 9234-0457
e-mail: ca.no.ne@hotmail.com

ORIENTADORA: ***Melania Maria Ramos de Amorim***
Doutora em Tocoginecologia pela Universidade de Campinas (UNICAMP)
Professora do Mestrado em Saúde Materno-Infantil do IMIP
RG: 937.512 – SSP/PB CPF: 569.821.784 – 04
Telefones: (081) 3221-5159/3221-0681 (083) 8815-5216
e-mail: melamorim@uol.com.br

CO-ORIENTADORA: ***Leila Katz***
Doutora em Tocoginecologia pela UNICAMP
Coordenadora da Unidade de Terapia Intensiva (UTI) obstétrica do IMIP
Preceptora da Residência Médica em Tocoginecologia do IMIP
RG: 2139813 SSP-PE CPF: 848225204-63
Telefones: (081) 88585977 (081) 21224739
e-mail: katzleila@gmail.com

CO-ORIENTADORA: ***Isabela Cristina Coutinho de Albuquerque Neiva Coêlho***
Doutora em Medicina pela Universidade Federal de Pernambuco
Preceptora da Residência Médica em Tocoginecologia do IMIP
RG: 3049471 SSP-PE CPF: 685531614-04
Telefones: (081) 3228-1289 3221-0681/5159 (081) 9272-7051
e-mail: isabela@elogica.com.br

COLABORADORA: ***Sabina Bastos Maia***
Mestranda em Cuidados Intensivos do IMIP
Médica Residente em Tocoginecologia do IMIP
RG: 2203593 SSP-PI CPF: 003.568.853-05
Telefone: (81) 9793-5117
e-mail: sabinamaia@hotmail.com

____
Assinatura

INSTITUIÇÃO: (Instituto de Medicina Integral Prof. Fernando Figueira - IMIP)

RESUMO

Introdução: As síndromes hipertensivas da gestação permanecem como importantes causas de morbimortalidade materna no cenário mundial. Na prática, estudos sobre pré-eclâmpsia costumam abordar o período pré-parto deixando o puerpério com abordagens restritas. Há, portanto, a necessidade de se conhecer melhor as alterações fisiopatológicas características deste período, principalmente a evolução dos níveis pressóricos e suas repercussões. **Objetivos**: Avaliar a efetividade da clonidina para tratamento hipotensor de emergência em puérperas com síndromes hipertensivas da gestação. **Métodos**: ensaio clínico, randomizado, triplamente mascarado, droga-controlado será realizado na Unidade de Terapia Intensiva obstétrica do Instituto de Medicina Integral Prof. Fernando Figueira no período de agosto a novembro de 2012. Serão incluídas 70 puérperas, com síndrome hipertensiva da gestação, apresentando pico hipertensivo, as quais serão randomizadas em dois grupos: captopril e clonidina. Puérperas com doenças maternas associadas, tabagistas, em uso de outras medicações hipotensoras ou drogas ilícitas, com contraindicações ao uso do captopril ou da clonidina. Os desfechos primários serão: pressão arterial sistólica, diastólica e média; frequência cardíaca e diminuição da frequência de picos hipertensivos. As variáveis referentes aos exames laboratoriais na admissão em unidade de terapia intensiva obstétrica do IMIP e sua evolução durante o puerpério; frequência de efeitos adversos da clonidina; necessidade de associação com outra medicação hipotensora; desfechos puerperais e complicações relacionadas aos picos hipertensivos serão considerados desfechos secundários. Após inclusão da puérpera no estudo, a caixa numerada (captopril ou clonidina) será disponibilizada a paciente com as devidas explicações sobre a forma de administração por via oral. A dose de captopril será de 25mg/dia,

podendo chegar até 150mg/dia, enquanto que a de clonidina será de 0,1mg/dia, com máximo de 0,6mg/dia a depender da recorrência de novos picos hipertensivos. A análise estatística será realizada utilizando o programa estatístico Epi-Info 7 e Minitab, versão 14.2. para Windows. A análise será realizada com os grupos identificados como A ou B, quebrando-se o sigilo somente depois de obtidos os resultados e preparadas as tabelas ou através da deliberação do Comitê de Monitorização Externa. As variáveis categóricas serão comparadas em tabelas de contingência, utilizando-se os testes Chi-quadrado de associação e exato de Fisher, quando pertinentes. Calcular-se-á a razão de risco (RR) como medida de risco relativo, determinando seu intervalo de confiança a 95%. Em relação às variáveis quantitativas, caso apresentem distribuição normal, a comparação entre os grupos será realizada através do teste *t de Student* para amostras não pareadas. Caso se verifique que a distribuição não é normal, utilizar-se-á o teste não paramétrico de *Mann-Whitney*.

Palavras-chaves: captopril, clonidina, período pós-parto, hipertensão induzida pela gestação, pré-eclâmpsia

SUMÁRIO

	Página
I. INTRODUÇÃO	1
II. JUSTIFICATIVA	15
III. HIPÓTESES	17
3.1. Hipóteses primárias	17
3.2. Hipóteses secundárias	17
IV. OBJETIVOS	18
4.1. Objetivo geral	18
4.2. Objetivos específicos	18
4.2.1. Primários	18
4.2.2. Secundários	18
V. MÉTODOS	20
5.1. Desenho do estudo	20
5.2. Local do estudo	20
5.3. Período de coleta de dados	21
5.4. População do estudo	21
5.5. Amostra	21
5.6. Tamanho amostral	22
5.7. Critérios para seleção dos Sujeitos	22
5.7.1. Critérios de inclusão	22
5.7.2 Critérios de exclusão	22

5.8. Variáveis 23

5.8.1. Variáveis Descritivas 23

5.8.2. Variáveis de Análise 24

5.8.2.1. Variáveis Dependentes 24

5.8.2.2. Variável Independente 27

5.9. Definição de Termos e Variáveis 27

5.9.1. Termos adotados 27

5.9.2. Critérios de Inclusão 28

5.9.3. Critérios de Exclusão 28

5.9.4. Variáveis do Estudo 29

5.10. Procedimentos para Seleção, Randomização e Acompanhamento dos Sujeitos 37

5.10.1. Procedimentos para Randomização dos Sujeitos 37

5.10.2. Procedimentos para preparo e administração das medicações 38

5.10.3. Procedimentos para Seleção dos Sujeitos 39

5.10.4. Acompanhamento dos Sujeitos 40

5.11. Caracterização Amostral 44

5.12. Procedimentos, Testes, Técnicas e Exames 44

5.13. Procedimentos para Coleta de Dados 48

5.13.1. Instrumento para coleta de dados 48

5.13.2. Coleta de Dados 49

5.13.3. Controle de qualidade das informações 49

5.14. Critérios para Descontinuação do Estudo 49

5.15. Processamento e Análise dos Dados 54

5.15.1. Processamento dos dados 54

5.15.2. Análise dos dados 55

5.16. Aspectos éticos 56

VI. RESULTADOS (*DUMMY TABLES*) 59

VII. CRONOGRAMA 69

VIII. ORÇAMENTO 70

IX. REFERÊNCIAS 73

APÊNDICE 1 - Aviso aos obstetras do Centro de Atenção a Mulher (CAM-IMIP) 79

APÊNDICE 2 - Lista de checagem 80

APÊNDICE 3 - Termo de consentimento livre e esclarecido 81

APÊNDICE 4 – Formulário 85

LISTA DE SIGLAS E ABREVIATURAS

ALT	Alanina transferase
AST	Aspartato transferase
AVC	Acidente vascular cerebral
bpm	batimento por minuto
CAM	Centro de atenção à mulher
CEP	Comitê de ética em pesquisa
CIVD	Coagulação intravascular disseminada
CD	*Compact disk*
CME	Comitê de monitorização externa
DHL	Desidrogenase lática
DP	Desvio padrão
DPPNI	Descolamento prematuro de placenta normoinserida
DUM	Data da última menstruação
EAP	Edema agudo de pulmão
FC	Frequência cardíaca
g	Grama
GABA	ácido g-aminobutírico
h	Hora
HELLP	Hemólise, plaquetopenia, elevação das enzimas hepáticas
HLA	*Human Leukocyte Antigens*
Id	Idade
IECA	Inibidor da enzima de conversão da angiotensina

IMC	Índice de massa corpórea
IMIP	Instituto de Medicina Integral Prof. Fernando Figueira
mg	Miligrama
ml	Mililitro
mm^3	milímetro cúbico
mg/dia	miligrama por dia
mg/dl	miligrama por decilitro
mmHg	milímetro de mercúrio
mEq/l	miliequivalente por litro
$MgSO_4$	Sulfato de magnésio
n	Amostra
NNT	Número necessário para tratar e obter um benefício
NNH	Número necessário para tratar e obter um malefício
p	Coefíciente estatístico
PA	Pressão arterial
PAD	Pressão arterial diastólica
PAM	Pressão arterial média
PAS	Pressão arterial sistólica
PE	Pernambuco
RR	Risco relativo
RCT	Ensaio clínico randomizado
SNC	Sistema nervoso central
SDS	Secretaria de defesa social
SSP	Secretaria de segurança pública

SPSS	Programa estatístico
SRA	Sistema renina angiotensina
SUS	Sistema Único de Saúde
TCLE	Termo de consentimento livre e esclarecido
UPE	Universidade de Pernambuco
UNICAMP	Universidade de Campinas
UTI	Unidade de Terapia Intensiva
UNIFESP	Universidade Federal de São Paulo
U/l	Unidades por litro
µg	Micrograma
µg/kg	micrograma por quilo
α_2-adrenérgico	alfa dois adrenérgico
+	uma cruz
%	Percentagem
$<$	Menor
$>$	Maior
$\leq$	menor ou igual
$\geq$	maior ou igual

ÍNDICE DE TABELAS

	Página
**Tabela 1 –** Média da idade materna, paridade, número de gestações e idade gestacional das puérperas com síndromes hiperensivas da gestação, apresentando pico hipertensivo, no momento da admissão em UTI obstétrica do IMIP.	59
**Tabela 2** – Freqüência cardíaca e pressão arterial das puérperas com síndromes hiperensivas da gestação, apresentando pico hipertensivo, no momento da admissão em UTI obstétrica do IMIP.	60
**Tabela 3** – Exames laboratoriais das puérperas com síndromes hiperensivas da gestação, apresentando pico hipertensivo, no momento da admissão em UTI obstétrica do IMIP.	61
**Tabela 4** – Exames laboratoriais nas puérperas com síndromes hiperensivas da gestação, apresentando pico hipertensivo, durante o internamento em UTI obstétrica do IMIP	62
**Tabela 5 –** Efeitos adversos em puérperas com síndromes hiperensivas da gestação, apresentando pico hipertensivo, durante o internamento em UTI obstétrica do IMIP	63
**Tabela 6 –** Médias do tempo de internamento em UTI obstétrica e idade gestacional do parto nas puérperas com síndromes hiperensivas da gestação, apresentando pico hipertensivo, durante o internamento em UTI obstétrica do IMIP	65
**Tabela 7** – Avaliação da efetividade da clonidina nas puérperas com	66

síndromes hiperensivas da gestação, apresentando pico hipertensivo, durante o internamento em UTI obstétrica do IMIP

Tabela 8 – Complicações maternas em puérperas com síndromes hiperensivas da gestação, apresentando pico hipertensivo, durante o internamento em UTI obstétrica do IMIP 67

Tabela 9 – Pressão arterial e freqüência cardíaca materna em puérperas com síndromes hiperensivas da gestação, apresentando pico hipertensivo, durante o internamento em UTI obstétrica do IMIP 68

ÍNDICE DE FIGURAS

Página

Figura 1 – Procedimentos para seleção e acompanhamento dos sujeitos 41

Figura 2 – Procedimentos para seleção e acompanhamento dos sujeitos (Fluxograma CONSORT) 43

Figura 3 – Fluxograma acompanhamento das pacientes. 46

Figura 4 – Fluxo das reuniões do Comitê de Monitorização Externa (CME) 53

Figura 5 – Curva de Sobrevida (análise de Kaplan-Meyer) para evolução dos níveis tensionais em puérperas usando captopril ou clonidina 68

1. INTRODUÇÃO

As síndromes hipertensivas da gestação permanecem como importantes causas de morbimortalidade materna no cenário mundial. Apesar dos esforços dispensados à prevenção, ao diagnóstico e o tratamento da hipertensão na gravidez, estima-se que mais de 60 mil mulheres morram por ano em decorrência deste agravo.[1]

De acordo com o *National High Blood Pressure Education Program Working Group on High Blood Pressure in Pregnancy* (2000), essas síndromes podem ser classificadas em hipertensão gestacional e crônica, pré-eclâmpsia leve, grave e superposta e eclâmpsia.[2] É importante ressaltar que dentro desta classificação a pré-eclâmpsia se destaca como a entidade clínica mais pesquisada e capaz de promover graves repercussões materno-fetais, estando a sua etiologia ainda por ser desvendada.[3,4]

Estudos de base hospitalar sugerem a existência de alguns aspectos imunogenéticos que influenciam a fisiopatologia da pré-eclâmpsia.[5] Aventa-se uma possível atuação do gene de síntese do óxido nítrico e do sistema *Human Leukocyte Antigens* (HLA) na resposta materna anormal ao trofoblasto.[5-7] Deste modo, lesões em camada endotelial vascular com ativação concomitante dos mecanismos de coagulação resultam na formação de microtrombos que levam ao comprometimento da perfusão tecidual sistêmica.[8,9]

Esses processos, em conjunto, ocasionam vasoespasmo sistêmico e hemoconcentração com redução na perfusão e acúmulo de líquido para o território extravascular (terceiro espaço). Portanto, as gestantes com pré-eclâmpsia na verdade são hipovolêmicas e a elevação da pressão arterial pode ser considerada uma consequência do mecanismo fisiopatológico da pré-eclâmpsia e não uma causa.[5]

De forma setorial, esses transtornos promovem um desequilíbrio entre fatores pró-angiogênicos e anti-angiogênicos na placenta resultando também em prejuízo vascular. A partir de um processo que leva à isquemia do anexo, a instalação dos quadros de pré-eclâmpsia se torna evidente. Admite-se que a criação de um ambiente intrauterino de alta resistência na camada muscular das arteríolas espiraladas favorece a redução do fluxo sanguíneo no leito placentário promovendo o surgimento e/ou manutenção de um processo de restrição de crescimento fetal, diminuição do volume de líquido amniótico e, em casos mais graves, óbito fetal.[10]

Conceitualmente, a pré-eclâmpsia é uma condição clínica que cursa com hipertensão e proteinúria ocorrendo depois da 20ª semana de gravidez. Esse distúrbio acomete 2% a 8% das gestações. Casos precoces podem surgir tendo como possíveis etiologias a doença trofoblástica gestacional ou a hidropsia fetal.[3]

Complicações potencialmente letais como o descolamento prematuro de placenta normoinserida (DPPNI), acidentes vasculares cerebrais (AVC), edema agudo de pulmão (EAP), falência hepática e renal e coagulação intravascular disseminada (CIVD) podem advir de um agravamento do quadro clínico.[4] Vale a pena salientar, que a subdivisão em pré-eclâmpsia leve e grave parte do princípio evolutivo da doença.

Os critérios de gravidade da pré-eclâmpsia podem ser evidenciados a partir da presença de um ou mais desses achados: pressão arterial sistólica (PAS) maior ou igual a 160mmHg, diastólica (PAD) maior ou igual a 110mmHg (ou ambos); proteinúria de dois gramas em 24 horas (3+ ou mais em exame qualitativo); nível sérico de creatinina maior que 1,2mg/dl; contagem de plaquetas menor que 100.000/mm^3 ou evidência de anemia microangiopática; elevação de enzimas hepáticas; persistência de cefaléia ou outros distúrbios cerebrovisuais e persistência de dor epigástrica.[3]

Na prática, estudos sobre pré-eclâmpsia costumam abordar o diagnóstico precoce e a identificação de seus fatores de risco com a intenção de promover a prevenção do agravo e impedir as consequências desfavoráveis ao binômio mãebebê. Assim, o período pré-parto é mais estudado na atualidade.[9]

Entretanto, a necessidade de se conhecer melhor o puerpério fica evidente ao se considerar as alterações fisiopatológicas características deste período. Um exemplo interessante seria o efeito da autotransfusão que ocorre no puerpério imediato, após a expulsão da placenta, quando é estimado um retorno abrupto de mais de 800ml de sangue à circulação materna. Essas e outras adaptações a que o organismo materno é submetido nesse período podem potencializar muitas das complicações da pré-eclâmpsia pós-parto, pouco pesquisadas.[9]

Um estudo de caso controle recente, incluindo 51 puérperas com pré-eclâmpsia, avaliou os fatores de risco para readmissão hospitalar nesse grupo de pacientes. Após análise criteriosa, mulheres afroamericanas, índice de massa corpórea (IMC) acima de 30, antecedente de síndromes hipertensivas e interrupção da gestação por cesárea foram os fatores que permaneceram associados ao desfecho. A raça asiática foi considerada protetora para impedir novas admissões de puérperas com pré-eclâmpsia em unidades hospitalares.[11]

A presença, no puerpério, de marcadores metabólicos para dislipidemias na pré-eclâmpsia foi avaliada em estudo observacional analítico também recente. Um total de 33 puérperas com pré-eclâmpsia e 200 mulheres sem complicações foram recrutadas. Após 24h-48h do parto, encontraram-se altas taxas de triglicérides ($p<0,01$), ácidos graxos livres ($p=0,02$), apolipoproteína B ($p=0,01$) e lipoproteína de alta densidade no grupo de puérperas com pré-eclâmpsia quando comparado ao grupo de mulheres

assintomáticas, demonstrando haver uma associação desse agravo com distúrbios metabólicos.[12]

Quanto às complicações sistêmicas inerentes à pré-eclâmpsia grave, a frequência encontrada em um estudo observacional realizado em Unidade de Terapia Intensiva (UTI) obstétrica do Recife foi considerada baixa (64/154), já que houve 43 episódios de oligúria (27,9%), um caso de EAP (0,6%), dois casos de eclâmpsia (1,3%) e 18 (11,7%) de síndrome HELLP. Há que se considerar a administração da terapia anticonvulsivante com sulfato de magnésio em todas as puérperas. Os achados estiveram de acordo com a frequência esperada de eclâmpsia descrita no Magpie (0,8%).[9,13]

A oligúria foi observada em 27,9% das puérperas, porém todos os episódios reverteram apenas com reposição volêmica, sem necessidade de diálise. A frequência observada pôde possivelmente ser justificada pelo jejum prolongado nas pacientes em uso de sulfato de magnésio, apesar da hidratação administrada de acordo com a rotina do serviço. Além da possibilidade de oligúria secundária à desidratação, a diminuição da diurese pode refletir a vasoespasticidade difusa da pré-eclâmpsia. Essa associação foi sugerida em um trabalho em que a duração da profilaxia anticonvulsivante com sulfato de magnésio poderia ser determinada pelo aumento do volume de diurese, refletindo uma provável resolução do vasoespasmo.[9,14]

Sabe-se que hipertensão, proteinúria e alterações bioquímicas causadas pela pré-eclâmpsia podem persistir de seis a 12 semanas. Os cuidados dispensados nos primeiros seis meses pós-parto devem incluir o manejo correto dos níveis pressóricos e a prevenção das possíveis causas secundárias ao período em que a paciente permanece hipertensa. Embora tenha sido postulado que esse tempo poderia ser curto para provocar lesões de órgãos-alvo, relatos da literatura evidenciaram a presença de distúrbios renais e persistência do quadro hipertensivo em avaliações prognósticas posteriores.[15]

O acompanhamento pós-parto ideal ainda não está determinado, porém os casos precisam ser individualizados. O aumento do risco de desenvolver doenças cardíacas no futuro é real e mulheres com pré-eclâmpsia sabidamente já possuem algum comprometimento metabólico prévio que só será diagnosticado a *posteriori*. Portanto, esse grupo particular de pacientes deve adotar os *guidelines* mais atuais de manutenção da saúde cardiovascular.[15]

Confirmando a hipótese de aumento no risco cardiovascular de forma aguda ou em longo prazo, um estudo epidemiológico americano demonstrou forte associação com pré-eclâmpsia e puerpério. Segundo os autores da pesquisa, as complicações cardiovasculares ocorrem em até 6% dos casos e a probabilidade de hipertensão arterial crônica, isquemia miocárdica, miocardiopatias dilatadas e disfunção cardíaca grave é considerável.[16]

Na evolução dos níveis tensionais em curto prazo, observa-se um padrão de comportamento uniforme no puerpério, tanto para os níveis máximos sistólicos quanto diastólicos. Depois de níveis elevados nos primeiros dias pós-parto, verifica-se uma redução estatisticamente significante dos níveis pressóricos diários, a partir do terceiro dia para os níveis sistólicos e a partir do quarto dia para os níveis diastólicos, até uma estabilização desses em cerca de quatro dias após o início da redução.[17] Nessas pacientes, a diminuição dos níveis pressóricos poderia ser justificada pela resolução do processo de lesão endotelial/vasoespasmo após o parto, com a eliminação do líquido extravasado para o terceiro espaço através da diurese, normalmente de grande volume nesse período. Entretanto, estudos avaliando o comportamento da diurese no puerpério para melhor fundamentação desta hipótese ainda são escassos.[9]

Em coorte retrospectiva envolvendo pacientes normotensas que desenvolveram hipertensão ou pré-eclâmpsia na gravidez, 81% delas normalizaram seu níveis

pressóricos em até três meses do parto, enquanto três (duas com hipertensão renovascular e uma com hiperaldosteranismo primário) permaneceram hipertensas por um período maior que seis meses.[18]

Quanto à evolução em longo prazo, acredita-se que o risco de desenvolver hipertensão em dois anos é alto, assim como a persistência de alterações funcionais/geométricas do ventrículo esquerdo. As implicações cardiovasculares da pré-eclâmpsia não acabam com o parto, nem com a retirada da placenta. A maior parte das puérperas com pré-eclâmpsia de surgimento precoce encontram-se no estágio B da insuficiência cardíaca e 40% desenvolvem hipertensão de um a dois anos após o parto.[16]

Uma das complicações mais comuns da hipertensão é a crise hipertensiva, que se caracteriza por elevação sintomática da pressão arterial (PA), com ou sem envolvimento de órgãos-alvo, podendo conduzir a um risco imediato ou potencial de vida. Sabe-se que a crise hipertensiva pode se manifestar em caráter de urgência ou emergência. Na emergência, há rápida deterioração de órgãos-alvo e risco imediato de vida, situação que não ocorre na urgência hipertensiva. Além disso, as situações em que a paciente apresenta PA elevada diante de algum evento emocional, doloroso ou desconfortável, sem evidências de lesões de órgãos-alvo ou risco imediato de vida, podem ser caracterizadas como pseudocrise hipertensiva, condição em que não é necessário o uso da terapia anti-hipertensiva de emergência.[19]

Segundo o último consenso de cardiologia,[13] o tratamento da emergência hipertensiva deve ser realizado com infusão venosa de nitroprussiato de sódio ou labetalol. O labetalol, embora não comercializado no Brasil, é a droga de escolha para o tratamento da hipertensão associada à pré-eclâmpsia. Nas urgências hipertensivas, a administração oral de nifedipina *retard* ou captopril é recomendada como primeira escolha terapêutica. Por outro lado, nas pseudoemergências hipertensivas, como não há

consequências orgânicas graves de forma imediata, as drogas hipotensoras podem ser administradas por via oral com redução dos níveis pressóricos em um prazo de até 24 horas.[20]

Não é só a hipertensão que mata as puérperas com pré-eclâmpsia. Em países desenvolvidos, 57% dos óbitos maternos são por eclampsia, tendo como causa básica a hemorragia intracraniana. A ruptura hepática associada à hemorragia pós-parto também são outras causas de morte neste grupo de pacientes.[17] Nos países em desenvolvimento, a falta de assistência adequada às puérperas com pré-eclâmpsia ou a sua evolução desfavorável faz dessa doença a maior responsável pela mortalidade materna nos países da América Latina e Caribe, incluindo o Brasil.[21]

Mesmo a partir dos conhecimentos adquiridos, ainda são escassos os estudos referentes à evolução dos níveis tensionais na puérpera com pré-eclâmpsia, bem como sua melhor forma de tratamento. Vale a pena lembrar que em alguns casos a simples elevação da PA não se constitui uma crise hipertensiva e não requer abordagem terapêutica imediata. O tratamento hipotensor agressivo pode trazer riscos para as pacientes, como a redução brusca dos níveis pressóricos favorecendo um quadro de hipotensão grave, isquemia do músculo cardíaco e alterações renais.[19]

A importância de se vigiar, tratar e observar picos hipertensivos tem sido destacada na obstetrícia, porém o tema é pouco abordado. Um estudo observacional realizado em UTI obstétrica evidenciou que mais da metade das pacientes com pré-eclâmpsia grave apresentaram picos pressóricos com necessidade de hipotensores no período pós-parto.[9]

Nesse mesmo estudo, analisando-se a alta hospitalar em uso de drogas hipotensoras, apenas 23,5% das pacientes saíram do hospital sem medicação, enquanto 76,5% receberam alta em uso de hipotensores. Quanto ao número de drogas utilizadas, a

frequência foi bem distribuída com 26,8%, 25,5% e 24% das pacientes usando, respectivamente, uma, duas ou três drogas no momento da alta. Apesar de alguns estudos avaliarem o uso de diuréticos e beta-bloqueadores para controle tensional no puerpério, estudos de boa qualidade analisando terapia anti-hipertensiva na pré-eclâmpsia são raros e com casuística escassa. Em uma revisão sistemática sobre prevenção e tratamento de hipertensão pós-parto, os revisores concluíram que não há dados suficientes para orientar a conduta nestas situações e que a experiência individual do médico assistente deve ser considerada.[9,22]

Em contrapartida, um ensaio clínico randomizado comparando a segurança e eficácia da administração venosa do labetalol e da hidralazina, na emergência hipertensiva no puerpério, evidenciou boa resposta pressórica, com redução de seus níveis e melhora dos sintomas associados, neste grupo particular de pacientes.[23]

A partir desta preocupação com o hipotensor que deverá ser utilizado no puerpério, as bases farmacológicas dos diversos agentes hipotensores utilizados nos distúrbios hipertensivos da gravidez, em especial na pré-eclâmpsia, foram revisadas. Os ensaios clínicos, fundamentados em estudos experimentais, têm evidenciado poucas categorias de fármacos que podem ser usados com segurança no ciclo grávido-puerperal.[24] A experiência mundial aponta hidralazina, nifedipina e labetalol como drogas de primeira escolha para controle da crise hipertensiva em gestantes. Metildopa continua sendo a droga de eleição para o tratamento da manutenção na gravidez. Hidralazina, nifedipina e verapamil são úteis também no tratamento de manutenção especialmente quando associados à metildopa. Os beta-bloqueadores podem também ser associados a um vasodilatador, embora possam provocar bradicardia fetal e neonatal. Embora exista um ensaio clínico que não evidencia resposta hemodinâmica

desfavorável com baixas doses de captopril,[25] os inibidores da enzima de conversão de angiotensina (IECA) estão contraindicados na gestação,[25] mas são drogas seguras após o parto e durante a lactação.[26]

O captopril (IECA) foi introduzido para uso clínico na hipertensão arterial nos anos 80. O efeito essencial deste agente sobre o sistema renina-angiotensina (SRA) é inibir a conversão da angiotensina I, relativamente inativa, em angiotensina II, forma ativa. Assim, são abolidas ou atenuadas as respostas à angiotensina com a diminuição da resistência vascular sistêmica e de PAS, PAD e PAM.[27]

A angiotensina II é um vasoconstritor extremamente poderoso. A vasoconstrição ocorre rapidamente, sendo muito intensa nas arteríolas e menos acentuada nas veias. A constrição das arteríolas aumenta a resistência vascular periférica elevando a PA. A constrição das veias aumenta o retorno venoso. Outro mecanismo importante para elevação da PA é sua ação renal, diminuindo a excreção de sal e água e aumentando lentamente o volume extracelular.[28]

Em revisão da literatura, utilizando as bases de dados Scopus, Cochrane, Lilacs e Pubmed, localizaram-se estudos conduzidos com a intenção de assegurar a efetividade e a segurança do captopril no tratamento dos picos pressóricos, inicialmente em pacientes hipertensos e depois no ciclo grávido-puerperal. Embora não sejam recentes, os dados disponíveis demonstraram, em geral, uma boa resposta do fármaco na hipertensão, ao passo que ações importantes na remodelação do músculo cardíaco pós-isquemia e nas situações de insuficiência deste órgão foram também relatadas.

Um ensaio clínico latino-americano demonstrou que 25mg de captopril sublingual

foi eficaz na redução dos níveis pressóricos em urgências hipertensivas quando comparado ao grupo que recebeu placebo. Segundo a farmacocinética da droga, existem dois períodos de absorção, um sublingual e o outro digestivo. Nesse estudo, a via sublingual foi escolhida porque garantiria níveis séricos da medicação de forma rápida e eficaz, com redução significativa da PA.[29] No entanto, alguns efeitos indesejáveis foram logo descritos, como é o caso das úlceras em mucosa oral e o sabor desagradável (amargo) referido pelos pacientes. Atualmente, a droga é administrada via oral com a mesma efetividade da sublingual e com menos efeitos colaterais.[30]

Quanto à associação com a nifedipina, estudo de intervenção americano selecionou 40 pacientes em unidades de emergência, com crise hipertensiva e cujos níveis tensionais não foram reduzidos de modo satisfatório após 20mg de flurosemida intravenosa. Esses pacientes foram subdivididos em dois grupos. Os pacientes do grupo A receberam 10mg de nifedipina via sublingual e os do grupo B 50mg de captopril via oral. Essas drogas foram repetidas com um intervalo de 30 minutos até um máximo de três doses ou até a obtenção de uma PAD $\leq$ 115mmHg. A maioria dos pacientes (77,5%) teve a sua PA reduzida aos níveis propostos após a primeira dose e o restante (22,5%) somente respondeu à terapêutica após a administração de doses subsequentes. Em nenhum caso foi observado efeito colateral, e todos os pacientes receberam alta da emergência sendo encaminhados para controle ambulatorial. Portanto, tanto a nifedipina quanto o captopril se mostraram eficazes para o tratamento da crise hipertensiva.[31]

O uso do captopril em pacientes com pré-eclâmpsia foi testado em um ensaio clínico aberto com 79 pacientes apresentando diagnóstico de pré-eclâmpsia grave e eclâmpsia. Dessas, 71 receberam captopril na dose de 25mg, com intervalo de 30 minutos em até três tomadas e as oito restantes receberam hidralazina intravenosa na

dose de 5mg, máximo de quatro doses com intervalo de 20 minutos. Das pacientes que receberam o captopril como droga hipotensora, 52 (73,3%) responderam de forma satisfatória ao fármaco com redução de até 25% dos níveis pressóricos. Em relação à quantidade de doses administradas, 20 pacientes (38%) requereram uma dose, 26 (50%) duas doses e seis (11%), três doses. Não foram observados efeitos adversos importantes, nem alterações da diurese e níveis de creatinina no neonato. Diante desses achados, os autores recomendam o uso de captopril sublingual para o manejo da crise hipertensiva na gravidez.[32]

Apesar de os estudos anteriores demonstrarem efetividade do captopril no controle da emergência hipertensiva e da manutenção dos níveis pressóricos estáveis, efeitos colaterais foram relatados pelas puérperas como a tosse seca, hipotensão, redução da função renal entre outros.[33] A partir desses achados, novas classes de hipotensores começaram a ser administradas com a mesma finalidade, como é o caso dos α_2-agonistas.

A clonidina é um α_2-agonista de ação central que apresenta efeito hipotensor sistêmico por diminuir o tônus do sistema nervoso simpático, promovendo estabilidade hemodinâmica. Além de seu efeito hipotensor, há relatos que a clonidina diminui a pressão intraocular, promove sedação, ansiólise, diminui a produção salivar e a concentração de catecolaminas circulantes.[34]

O cloridrato de clonidina age relativamente rápido, com pico de concentração plasmática e efeito hipotensor máximo de uma a três horas após a administração oral. A eliminação da droga varia de seis a 24 horas, com média de 12 horas, e em crianças a meia-vida parece ser menor (1,8 a 2,4 horas). Boca seca e sedação ocorrem em 50% dos pacientes com o uso da clonidina como hipotensor. Os efeitos colaterais mais

comumente descritos na literatura são sonolência (35%), tontura (35%) e hipotensão arterial (5%).[35]

Vale a pena salientar que o cloridrato de clonidina não é disponível em suspensão, portanto erros de diluição podem causar intoxicação e até morte. Deve-se observar sempre a presença de sinais de intoxicação aguda, como sonolência precoce, miose e bradicardia. Casos de intoxicação por clonidina já foram descritos com doses tão baixas quanto 0,2mg. A administração de volume para hipotensão, atropina para bradicardia e naloxone para depressão respiratória revertem geralmente o quadro. Por isso, na prática diária deve-se utilizar a medicação comercial e não a manipulada, com maceração do comprimido e diluição em água destilada para a correta administração da dose prescrita.[35]

Na posição supina, quando o tônus simpático para a vasculatura é baixo, o efeito maior da clonidina é diminuir tanto a frequência cardíaca quanto o volume sistólico. Entretanto, na posição ortostática, quando o fluxo simpático para a vasculatura está normalmente aumentado, a droga reduz a resistência vascular. Algum grau de hipotensão ortostática sempre ocorre por causa da diminuição do retorno venoso (secundário à venodilatação sistêmica). Porém, a hipotensão postural é rara na ausência de depleção de volume.[36]

Os efeitos respiratórios da clonidina têm sido bastante debatidos, mas o consenso é que os agonistas α_2-adrenérgicos estão associados com depressão respiratória mínima. Apesar de estarem presentes no cérebro, não possuem um papel no controle central da respiração.[36]

Estudos têm demonstrado a eficácia da clonidina como fármaco sedativo. Quando utilizada para sedação leve, a clonidina teve resultados comparáveis ao fentanil,

evitando doses extras de sedativos. Sabe-se que os receptores α_2-adrenérgicos têm parte de sua ação farmacológica através da ativação dos mesmos canais de potássio que os receptores opióides, o que poderia justificar o comportamento semelhante da clonidina em relação aos opióides no que diz respeito à sedação.[37]

O sítio para ação sedativa dos agonistas α_2-adrenérgicos se encontra no *locus coeruleus* do tronco cerebral e este, quando estimulado, leva à inibição da regulação do sono e da vigília. As principais vias noradrenérgicas ascendentes e descendentes originam-se dessa importante área. A ativação dos receptores α_2 no *locus coeruleus* produz supressão de sua atividade, resultando em aumento da atividade de interneurônios inibitórios, como a via do ácido g-aminobutírico (GABA), o que determina depressão do sistema nervoso central (SNC).[38]

O efeito da clonidina nas variáveis hemodinâmicas é controverso, com alguns autores sugerindo que não há alterações importantes em pacientes jovens e hígidos, enquanto outros relatam diminuição da PA sistêmica e bradicardia, mesmo nesse grupo.[39]

A clonidina, independentemente da dose administrada, apresenta início de ação e efeito clínico mais rápido que a nifedipina, permitindo, na prática, um bom e apurado controle clínico. Após a administração de clonidina venosa, pode ocorrer aumento da PA e diminuição do débito cardíaco nos primeiros dois minutos. A diminuição precoce dos valores pressóricos e da frequência cardíaca em pacientes que receberam clonidina está de acordo com o estudo da farmacocinética da clonidina por via venosa, que demonstrou que o tempo de distribuição da droga no organismo é em torno de 10 minutos.[40]

Esses dados demonstraram a maior eficácia da clonidina em relação à nifedipina no controle de crises hipertensivas. Entretanto, a dose de 3µg/kg de clonidina esteve associada com alta incidência de sonolência e diminuição da produção salivar. A sonolência obtida em 40% dos pacientes que receberam essa dose também pode dificultar o ato cirúrgico em cirurgia oftalmológica sob anestesia local, devido à possibilidade de obstruir parcialmente as vias aéreas superiores e, consequentemente, levar à movimentação da cabeça durante o esforço inspiratório. Já a diminuição da produção salivar pode provocar desconforto e irritação faríngea sendo necessário umedecer a orofaringe com pequenas quantidades de água.[40]

No ciclo grávido-puerperal, um ensaio clínico americano recrutou 82 gestantes com hipertensão para tratamento com clonidina no último trimestre da gestação. Os resultados demonstraram a efetividade da clonidina endovenosa como agente hipotensor em todos os grupos tratados: hipertensão essencial (n=31), pré-eclâmpsia leve (n=7), pré-eclâmpsia grave (n=7) e hipertensão crônica agravada pela gestação (n=24). A droga foi bem tolerada entre as mulheres e apenas um feto morreu por hipóxia intrautero no grupo de pacientes com pré-eclâmpsia grave, enquanto outro morreu após um dia do parto com sepse..[41]

Assim, se o tratamento hipotensor faz-se necessário durante a gravidez ou o puerpério e não há contraindicações ao uso de α_2-adrenérgicos, a clonidina pode ser uma opção interessante em emergências hipertensivas. Desta forma, diante das incertezas, os autores ressaltam a necessidade de um ensaio clínico randomizado que avalie a efetividade e os efeitos colaterais da clonidina para tratamento hipotensor de emergência no puerpério em comparação com o uso de captopril.

II – JUSTIFICATIVA

O puerpério representa uma fase do ciclo grávido-puerperal ainda pouco abordada cientificamente. Não há relatos suficientes e concretos na literatura que elucidem questões importantes, principalmente no campo da hipertensão e gravidez. Pesquisas baseadas em evidências atuais concentram seu enfoque no diagnóstico das síndromes hipertensivas e no tratamento das repercussões maternofetais desses agravos. No entanto, o prognóstico em curto e longo prazo, como a evolução da PA em puérperas com pré-eclâmpsia grave, o tratamento mais efetivo para o controle da crise hipertensiva e as manifestações cardiovasculares e metabólicas após dois anos da interrupção da gestação carecem de maiores esclarecimentos.

A idéia principal para o desenvolvimento desta pesquisa surgiu da experiência clínica do uso de clonidina para tratamento da emergência hipertensiva em puérperas com pré-eclâmpsia grave ou hipertensão crônica agravada pela gestação, internadas em UTI obstétrica do Instituto de Medicina Integral Prof. Fernando Figueira (IMIP), Recife/PE-Brasil. Esse fármaco começou a ser empregado em pacientes com alguma restrição ao uso dos IECA e o seu efeito hipotensor foi considerado satisfatório. O que não se tem conhecimento ainda é em quanto tempo a clonidina reduz os níveis pressóricos e por quanto tempo os deixa estabilizados em comparação ao uso de captopril, medicação preconizada para o tratamento agudo da crise hipetensiva, segundo as normas técnicas da instituição.

A coleta de dados deverá ser realizada na UTI obstétrica do IMIP que dispõe de 10 leitos com uma frequência de 87% de puérperas admitidas com síndromes hipertensivas, correspondendo a cerca de 1.000 internações anuais. Desta forma, caracteriza-se a facilidade em atingir o número total de participantes, visto que o IMIP é

referência no atendimento às gestantes de alto risco em Pernambuco e cidades circunvizinhas. A equipe de pesquisadores é especialista em tocoginecologia com experiência em terapia intensiva, apresentando a *expertise* necessária à condução dos trabalhos.

Esta pesquisa não oferecerá riscos potenciais para as puérperas visto que as medicações (captopril e clonidina) são de uso preconizado na hipertensão e fazem parte da rotina do serviço, assim como o manejo clínico e laboratorial não será alterado com a sua participação no estudo. A escolha em participar e permanecer no projeto será de cada paciente, mantendo-se assim a autonomia como um dos princípios da ética em pesquisa.

Os autores se comprometem a publicar o estudo em revista internacional com bom fator de impacto, mesmo que os resultados não sejam significantes ou discordantes dos encontrados na literatura, independente de favorecerem essa ou aquela droga.

III – HIPÓTESES

Em puérperas, com síndromes hipertensivas da gestação, fazendo uso de clonidina ou captopril para tratamento hipotensor de emergência:

3.1 – Hipóteses primárias

1. A pressão arterial (sistólica, diastólica e média) diminui e se mantém estável, reduzindo-se o risco de hipertensão não controlada com a utilização da clonidina;
2. A clonidina, quando comparada ao captopril, apresenta boa efetividade na diminuição da frequência de picos hipertensivos.

3.2 – Hipóteses secundárias

1. O uso da clondina, quando comparada ao captopril, não altera os exames laboratoriais durante o internamento em UTI obstétrica;
2. A frequência de efeitos colaterais dos dois hipotensores é similar aos descritos na literatura;
3. A necessidade de introdução de novos hipotensores é similar aos descritos na literatura;
4. Não há diferenças nos desfechos puerperais e nas complicações maternas.

IV - OBJETIVOS

4.1 – Objetivo Geral

Avaliar a efetividade da clonidina, em relação ao captopril, para tratamento hipotensor de emergência em puérperas com síndromes hipertensivas da gestação.

4.2 – Objetivos Específicos

Em puérperas, com síndromes hipertensivas da gestação, randomizadas para receber tratamento hipotensor de emergência com clonidina ou captopril, comparar:

4.2.1 - Desfechos Primários

1. A evolução dos parâmetros clínicos durante a internação em UTI obstétrica do IMIP: pressão arterial (sistólica, diastólica e pressão arterial média) e frequência cardíaca;
2. Frequência de picos hipertensivos nas primeiras 24 horas de uso.

4.2.2 - Desfechos Secundários

1. Exames laboratoriais na admissão em UTI obstétrica do IMIP e sua evolução durante o puerpério: proteinúria de fita, uréia, creatinina, ácido úrico, sódio, potássio e cloro, desidrogenase lática (DHL), aspartato transferase (AST), alanina transferase (ALT), bilirrubinas totais e frações e plaquetas;
2. Frequência de efeitos adversos: secura na boca, sensação de cansaço, distúrbio ortostático, vertigem, obstipação, transtornos do sono, distúrbios circulatórios, transtornos da percepção, pesadelo, dores nas glândulas parótidas, estado de depressão, secura da mucosa nasal e diminuição da lacrimação (clonidina).

Tosse seca, erupções cutâneas, prurido, febre, artralgia, lesão de pele tipo penfigóide, reações de fotossensibilidade, rubor ou palidez, hipotensão, taquicardia, dores no peito, palpitações, disgeusia, anemia, trombocitopenia, pancitopenia, angioedema e insuficiência renal (captopril).

3. Necessidade de associação com outra medicação hipotensora.
4. Desfechos puerperais: dias de internamento em UTI obstétrica, complicações maternas (eclâmpsia, síndrome HELLP, iminência de eclâmpsia, oligúria, complicações puerperais e óbito materno) e complicações relacionadas aos picos hipertensivos (AVC, infarto agudo do miocárdio e edema agudo de pulmão).

V. MÉTODO

5.1. Desenho do estudo

Será realizado um ensaio clínico, randomizado, droga-controlado e triplo cego. A medicação hipotensora para o pico hipertensivo utilizada no grupo controle será o captopril, rotineiramente empregado segundo protocolo do serviço.[42]

5.2. Local do Estudo

O estudo será desenvolvido na maternidade do IMIP, setor UTI Obstétrica localizada em Recife – Pernambuco/Brasil.

O IMIP é uma entidade filantrópica (sem fins lucrativos) que atende às comunidades carentes do Estado e regiões próximas. Credenciado pelos Ministérios da Educação e Saúde como hospital de ensino, mantém o título de centro de referência nacional e estadual na assistência Materno-Infantil segundo o Ministério da Saúde e Sistema Único de Saúde (SUS)-PE, respectivamente.

No setor de Maternidade, contabilizam-se anualmente cerca de 6.000 partos, com elevado percentual de puérperas consideradas de alto-risco (em torno de 50%). A instituição apresenta uma frequência geral de 12,5% de síndromes hipertensivas, que são responsáveis por 87% das admissões em UTI obstétrica.[43]

A UTI obstétrica do IMIP foi criada em março de 2002, com 10 leitos destinados à promoção da saúde das mulheres do Estado e cidades circunvizinhas. A equipe é formada por médicos tocoginecologistas, com experiência em terapia intensiva e gestações de alto risco, enfermeiros e técnicos de enfermagem devidamente habilitados

para prestarem os cuidados necessários às puérperas graves. Também fazem parte, indiretamente, do corpo clínico: médicos e enfermeiros residentes em Saúde da Mulher, outras especialidades (clínicos, cirurgiões, anestesistas, fisioterapeutas, fonoaudiólogos, psicólogos e nutricionistas), contribuindo para a assistência transdisciplinar prestada a esse grupo peculiar de pacientes.

5.3. Período de Coleta de dados

A coleta dos dados será realizada no período compreendido entre os meses de agosto de 2012 a novembro de 2012.

5.4. População do Estudo

Puérperas com síndromes hipertensivas da gestação, admitidas na UTI obstétrica do IMIP no período da pesquisa.

A idade materna média da população do estudo é de 25 anos, sendo aproximadamente 53% procedentes do Recife e Região Metropolitana e 47% de cidades do interior do estado de Pernambuco ou outros Estados. Em torno de 52% apresentam escolaridade entre zero e sete anos de estudo e 48% com oito ou mais anos completos. Em relação à paridade, 27% são primíparas e 38% multíparas.[43]

5.5. Amostra

De acordo com os critérios de inclusão e exclusão, serão selecionadas puérperas com síndromes hipertensivas da gestação, apresentando picos hipertensivos durante

intermamento no setor UTI obstétrica do IMIP. As puérperas elegíveis para o estudo serão randomizadas para receber, como terapia anti-hipertensiva de emergência, captopril ou clonidina.

5.6. Tamanho Amostral

Para este cálculo foi utilizado o programa Statcalc do software Epi-Info 7, sessão OpenEpi (Cohort/RCT). O cálculo foi realizado prevendo-se, para uma amostra com hipertensão, uma redução da frequência de picos hipertensivos após uso oral de captopril de 64% e após uso transdérmico de clonidina de 99%.[37] Obteve-se uma amostra de 52 puérperas para um erro alfa de 0,05 e um erro beta de 0,10.[44] Prevendo-se uma taxa de perdas de 20% serão selecionadas 70 sujeitos para a pesquisa, randomizadas para dois grupos (35 em uso de captopril e 35, clonidina).

5.7. Critérios para Seleção dos Sujeitos

5.7.1 – Critérios de Inclusão

- Puérperas;
- Síndromes hipertensivas da gestação;
- Pico hipertensivo.

5.7.2 – Critérios de Exclusão

- Doenças maternas associadas como diabetes Mellitus, colagenoses, glomerulonefrites, pneumopatias e cardiopatias;
- Tabagismo;

- Uso de outras medicações hipotensoras ou drogas ilícitas que possam interferir na hemodinâmica materna;
- Contraindicações ao uso do captopril: insuficiência renal, hepatopatias crônicas e hipersensibilidade ao uso da droga;
- Contraindicações ao uso da clonidina: doença do nódulo sinusal, hepatopatias crônicas e hipersensibilidade ao uso da droga.

5.8. Variáveis

5.8.1. Variáveis Descritivas

Característica biológica:

- Idade materna.

Características obstétricas:

- Idade gestacional do diagnóstico da síndrome hipertensiva da gestação;
- Idade gestacional na interrupção da gravidez;
- Número de gestações;
- Paridade;
- Hipertensão gestacional;
- Hipertensão crônica;
- Pré-eclampsia grave;
- Pré-eclampsia superposta.

Parâmetros clínicos puerperais no momento da admissão em UTI obstétrica do IMIP:

- Pressão arterial sistólica;
- Pressão arterial diastólica;

- Pressão arterial média;
- Frequência cardíaca;

Exames laboratoriais da puérpera no momento da admissão em UTI obstétrica do IMIP:

- Proteinúria de fita;
- Função renal: uréia, creatinina e ácido úrico;
- Função hepática: DHL, ALT, AST, bilirrubinas totais, direta e indireta;
- Contagem do número de plaquetas.

5.8.2. Variáveis de Análise

5.8.2.1. Variáveis Dependentes

- **Desfechos Primários**

Parâmetros clínicos puerperais:

- Pressão arterial sistólica;
- Pressão arterial diastólica;
- Pressão arterial média;
- Frequência cardíaca;

Efetividade da clonidina como droga hipotensora para o pico hipertensivo:

- Necessidade de associação com outro agente hipotensor;
- Necessidade de manutenção da terapia hipotensora para o pico hipertensivo;
- Frequência de picos hipertensivos nos primeiros cinco, 10, 15 e 20 minutos após uso inicial de clonidina;
- Frequência de picos hipertensivos após duas horas do uso inicial de clonidina;

- ❖ Frequência de picos hipertensivos nas 24 horas após uso inicial de clonidina.

- **Desfechos Secundários**

Exames laboratoriais da puérpera durante o internamento na UTI obstétrica do IMIP:

- ❖ Proteinúria de fita;
- ❖ Função renal: uréia, creatinina e ácido úrico;
- ❖ Ionograma: sódio, potássio e cloro;
- ❖ Função hepática: DHL, AST, ALT, bilirrubinas totais, direta e indireta;
- ❖ Contagem do número de plaquetas.

Efeitos adversos da clonidina[38]

- ❖ Secura da boca;
- ❖ Sensação de cansaço;
- ❖ Distúrbio ortostático;
- ❖ Vertigem;
- ❖ Obstipação;
- ❖ Transtornos do sono;
- ❖ Distúrbios circulatórios;
- ❖ Transtornos da percepção;
- ❖ Pesadelo;
- ❖ Dores das glândulas parótidas;
- ❖ Estado de depressão;
- ❖ Secura da mucosa nasal;
- ❖ Diminuição da lacrimação.

Efeitos adversos da captopril[32]

- ❖ Tosse seca;
- ❖ Erupções cutâneas;
- ❖ Prurido;
- ❖ Febre;
- ❖ Artralgia;
- ❖ Lesão de pele tipo penfigóide;
- ❖ Reações de fotossensibilidade;
- ❖ Rubor;
- ❖ Palidez;
- ❖ Hipotensão;
- ❖ Taquicardia;
- ❖ Dores no peito;
- ❖ Palpitações;
- ❖ Disgeusia;
- ❖ Anemia;
- ❖ Trombocitopenia;
- ❖ Pancitopenia;
- ❖ Angioedema;
- ❖ Insuficiência renal.

Complicações puerperais durante internamento em UTI obstétrica:

- ❖ Edema agudo de pulmão;
- ❖ Coagulação intravascular disseminada;
- ❖ Eclâmpsia;

- Síndrome HELLP;
- Iminência de eclampsia;
- Oligúria;
- Óbito materno;
- Complicações maternas relacionadas às síndromes hipertensivas da gravidez;
- Complicações maternas não relacionadas às síndromes hipertensivas da gravidez.

5.8.2.2. Variável Independente

- Uso de clonidina como hipotensor para o pico hipertensivo.

5.9. Definição de Termos, Variáveis e dos Critérios para Seleção dos Sujeitos

5.9.1. Termos Adotados

- Hipertensão – definida como aumento dos níveis hipertensivos, sendo $\geq$ 140 mmHg a sistólica e/ou $\geq$ 90 mmHg a diastólica, aferida por duas vezes consecutivas com intervalo maior que 30 minutos e após repouso em decúbito lateral esquerdo.[3,13]
- Proteinúria – a partir de 300 mg de proteínas na urina de 24 h ou proteinúria de fita de 2+ ou mais em amostra isolada ou 1+ em duas amostras consecutivas com intervalo de no mínimo 30 minutos.[3]
- Pré-eclâmpsia – hipertensão e proteinúria presentes acima da 20ª semana de gravidez.[3]
- Hipertensão grave – segundo os critérios do *Sétimo Report of the Joint National Committee on Prevention, Detection, Evaluation, and Treatment of High Blood Pressure* (2003), define o estágio dois (grave) da hipertensão arterial como sendo a pressão arterial sistólica $\geq$ 160mmHg e/ou diastólica $\geq$ 100mmHg.[13]

- Controle da pressão arterial – pressão arterial sistólica $\leq$ 170mmHg e diastólica $\leq$ 110mmHg, podendo ocorrer picos hipertensivos esporádicos (menor que três em 24 horas).[13]

5.9.2. Critérios de Inclusão

- Síndromes hipertensivas da gestação - serão incluídas as formas clínicas das síndromes hipertensivas que ocorrem na gestação, como: pré-eclâmpsia grave, hipertensão gestacional, hipertensão crônica, pré-eclâmpsia superposta, eclâmpsia, síndrome HELLP[3]
- Pico hipertensivo – pressão arterial sistólica $\geq$ 170mmHg e/ou diastólica $\geq$ 110mmHg.[13]
- Puerpério – fase do ciclo grávido-puerperal que se inicia após o parto e termina no 42º dia

5.9.3. Critérios de Exclusão

- Doenças maternas associadas – qualquer doença clínico-cirúrgica associada à gravidez, como insuficiência cardíaca, doenças auto-imunes, insuficiência renal, cardiopatias, pneumopatias, epilepsia e obesidade, segundo a curva do IMC elaborada no Chile[45] e utilizada pelo Ministério da Saúde do Brasil.[46]
- Tabagismo – definido como o hábito de fumar durante a gestação, independentemente do número de cigarros, no ínicio do estudo.
- Uso de outras medicações hipotensoras ou drogas ilícitas – uso de quaisquer medicações hipotensoras ou drogas ilícitas utilizadas *antes* do início da aplicação do protocolo desta pesquisa, exceto suplementação com vitaminas e ferro.

- Contraindicações ao uso do captopril – puérperas com conhecida contraindicação ao captopril, como hipersensibilidade a droga, insuficiência renal e hepatopatias crônicas.[47]
- Contraindicações ao uso da clonidina – puérperas com conhecida contraindicação a clonidina, como doença do nódulo sinusal, hepatopatias crônicas e hipersensibilidade ao uso da droga.

5.9.4. Variáveis do Estudo

- Idade materna – variável numérica, discreta, expressa em anos completos, conforme informação da puérpera.
- Idade gestacional - em semanas completas, calculada a partir da data da última menstruação (DUM), desde que conhecida e confiável, confirmada pelo primeiro exame ecográfico. Quando a puérpera não souber referir a DUM e/ou houver discordância com o exame ultrassonográfico, a datação da gestação será realizada com base na primeira ultrassonografia.
- Idade gestacional do diagnóstico da síndrome hipertensiva da gestação - Idade gestacional calculada tomando como base o dia do diagnóstico da síndrome hipertensiva da gestação. Variável numérica, contínua.
- Idade gestacional na interrupção da gestação – idade gestacional calculada tomando como base o dia do parto. Variável numérica, contínua.
- Número de gestações – variável numérica, discreta, correspondendo ao número de gestações, conforme informação da puérpera.[43]
- Paridade – variável numérica, discreta, correspondendo ao número de partos (conceptos pesando 500g ou mais), conforme informação da puérpera.[43]

- Pré-eclâmpsia grave – pressão sistólica ≥ 160 mmHg e/ou diastólica ≥ 110 mmHg com proteinúria; hipertensão associada com proteinúria grave; hipertensão associada a comprometimento de múltiplos órgãos; e hipertensão associada a sintomas persistentes (visuais, cerebrais e dor abdominal).[3] Variável nominal, dicotômica do tipo sim/não.
- Síndrome HELLP – associação nas puérperas com pré-eclâmpsia de achados laboratoriais característicos da síndrome HELLP: hemólise (bilirrubinas totais > 1,2 mg/dl e DHL > 600 U/L), elevação das enzimas hepáticas (TGO > 70 mg/dl e DHL > 600 U/L) e plaquetopenia (plaquetas < 100.000 mm^3).[3] Variável nominal, dicotômica do tipo sim/não.
- Iminência de eclâmpsia – presença, em puérperas com diagnóstico de pré-eclâmpsia, de alguns desses sinais e sintomas: cefaléia contínua, escótomas, turvação visual, dor epigástrica e dor em hipocôndrio direito.[3] Variável nominal, dicotômica do tipo sim/não.
- Eclâmpsia – pré-eclâmpsia associada a convulsões que não podem ser atribuídas a outras causas.[3] Variável nominal, dicotômica do tipo sim/não.
- Hipertensão crônica com pré-eclâmpsia superposta – definida como a presença de hipertensão crônica associada ao aumento súbito dos níveis hipertensivos e/ou da proteinúria ou evidência de alguma disfunção orgânica, através de sintomas de iminência de eclâmpsia ou alterações dos exames laboratoriais (plaquetas, uréia, creatinina, ácido úrico, AST, ALT, DHL, Bilirrubinas totais, direta e indireta).[3] Variável nominal, dicotômica do tipo sim/não.
- Hipertensão gestacional – segundo os critérios do *Sétimo Report of the Joint National Committee on Prevention, Detection, Evaluation, and Treatment of High Blood Pressure* (2003), define o estágio dois (grave) da hipertensão arterial como sendo a

pressão arterial sistólica $\geq$ 160mmHg e/ou diastólica $\geq$ 100mmHg.[13] Variável nominal, dicotômica do tipo sim/não.

- ❖ Pressão arterial sistólica (PAS) – valor da pressão arterial sistólica em milímetro de mercúrio, aferida no momento de admissão da puérpera e durante seu acompanhamento em UTI obstétrica do IMIP. Variável numérica, contínua.
- ❖ Pressão arterial diastólica (PAD) – valor da pressão arterial diastólica em milímetro de mercúrio, aferida no momento de admissão da puépera e durante seu acompanhamento em UTI obstétrica do IMIP. Variável numérica, contínua.
- ❖ Pressão arterial média (PAM) – valor da pressão arterial média em milímetro de mercúrio, aferida no momento de admissão da puépera e durante seu acompanhamento em UTI obstétrica do IMIP. Será calculada pela fórmula: pressão sistólica (PAS) mais duas vezes a pressão diastólica (PAD) dividido por três, sendo representada por (PAS + 2PAD)/3. Variável numérica, contínua.
- ❖ Frequência cardíaca – número de batimentos cardíacos por minuto, aferida pelo pulso no momento de admissão da puépera e durante seu acompanhamento em UTI obstétrica do IMIP. Variável numérica, discreta.
- ❖ Proteinúria de fita – variável categorizada em negativo, traços, uma, duas, três e quatro cruzes, correspondendo à presença de proteína na urina no momento da realização do exame.
- ❖ Uréia – exame laboratorial realizado no momento de admissão da puépera e durante seu acompanhamento em UTI obstétrica do IMIP, correspondente ao valor numérico em mg/dl no sangue, sendo considerado os valores normais abaixo de 15mg/dl. Variável numérica, contínua.
- ❖ Creatinina - exame laboratorial realizado no momento de admissão da puépera e durante seu acompanhamento em UTI obstétrica do IMIP, correspondente ao valor

numérico em mg/dl no sangue, sendo considerado os valores normais abaixo de 1,3mg/dl. Variável numérica, contínua.

- Ácido úrico - exame laboratorial realizado no momento de admissão da puépera e durante seu acompanhamento em UTI obstétrica do IMIP, correspondente ao valor numérico em mg/dl no sangue, sendo considerado os valores normais abaixo de 4mg/dl. Variável numérica, contínua.
- Sódio – exame laboratorial realizado no momento de admissão da puépera e durante seu acompanhamento em UTI obstétrica do IMIP, correspondente ao valor numérico em mEq/l no sangue, sendo considerado os valores normais de 135mEq/l a 145mEq/l. Variável numérica, contínua.
- Potássio - exame laboratorial realizado no momento de admissão da puépera e durante seu acompanhamento em UTI obstétrica do IMIP, correspondente ao valor numérico em mEq/l no sangue, sendo considerado os valores normais de 3,5mEq/l a 5,0mEq/l. Variável numérica, contínua.
- Cloro - exame laboratorial realizado no momento de admissão da puépera e durante seu acompanhamento em UTI obstétrica do IMIP, correspondente ao valor numérico em mEq/l no sangue, sendo considerado os valores normais de 98mEq/l - 106mEq/l. Variável numérica, contínua.
- Desidrogenase láctica (DHL) – exame laboratorial realizado no momento de admissão da puépera e durante seu acompanhamento em UTI obstétrica do IMIP, correspondente ao valor numérico em U/l no sangue, sendo considerado como valor normal menor que 600U/l. Variável numérica, contínua.
- Aspartato transaminase (AST) – exame laboratorial realizado no momento de admissão da puérpera e durante seu acompanhamento em UTI obstétrica do IMIP

correspondente ao valor numérico em U/l no sangue, considerando valor de referência de AST < 70U/l. Variável numérica, contínua.

- Alanina transaminase (ALT) - exame laboratorial realizado no momento de admissão da puérpera e durante seu acompanhamento em UTI obstétrica do IMIP correspondente ao valor numérico em U/l no sangue, considerando valor de referência de ALT < 50U/l. Variável numérica, contínua.
- Bilirrubina total – exame laboratorial realizado no momento de admissão da puépera e durante seu acompanhamento em UTI obstétrica do IMIP, correspondente ao valor numérico em mg/dl no sangue, sendo os valores de referência da bilirrubina total < 1,2mg/dl. Variável numérica e contínua.
- Bilirrubina direta - exame laboratorial realizado no momento de admissão da puépera e durante seu acompanhamento em UTI obstétrica do IMIP, correspondente ao valor numérico em mg/dl no sangue, sendo os valores de referência da bilirrubina direta < 0,4mg/dl. Variável numérica e contínua.
- Bilirrubina indireta - exame laboratorial realizado no momento de admissão da puépera e durante seu acompanhamento em UTI obstétrica do IMIP, correspondente ao valor numérico em mg/dl no sangue, sendo os valores de referência da bilirrubina indireta < 0,8mg/dl. Variável numérica e contínua.
- Plaquetas – exame laboratorial realizado no momento de admissão da puépera e durante seu acompanhamento em UTI obstétrica do IMIP, correspondente ao valor numérico de plaquetas por mm^3 no sangue, sendo normal o valor maior ou igual a 100.000$mm^{3.}$ Variável numérica, discreta.
- Secura da boca – ausência de salivação após iniciada após uso da medicação hipotensora, referida pela puérpera. Variável nominal, dicotômica do tipo sim/não.

- Distúrbio ortostático - presença de alteração da pressão arterial em ortostase alteração após iniciada após uso da medicação hipotensora, referida pela puérpera. Variável nominal, dicotômica do tipo sim/não.
- Obstipação – dificuldade ou ausência da eliminação das fezes após iniciada após uso da medicação hipotensora, referida pela puérpera. Variável nominal, dicotômica do tipo sim/não.
- Transtornos da percepção - presença dessas alterações após iniciada após uso da medicação hipotensora, referida pela puérpera. Variável nominal, dicotômica do tipo sim/não.
- Pesadelo – presença dessa alteração após iniciada após uso da medicação hipotensora, referida pela puérpera. Variável nominal, dicotômica do tipo sim/não.
- Dores das glândulas parótidas – dores em pescoço, abaixo da mandíbula, após iniciada após uso da medicação hipotensora, referida pela puérpera. Variável nominal, dicotômica do tipo sim/não.
- Estado de depressão – puérpera com queixa de falta de estímulo e de interesse pelo tratamento e sua vida após iniciada após uso da medicação hipotensora, referida pela puérpera. Variável nominal, dicotômica do tipo sim/não.
- Secura da mucosa nasal – presença dessa alteração após iniciada após uso da medicação hipotensora, referida pela puérpera. Variável nominal, dicotômica do tipo sim/não.
- Diminuição da lacrimação – diminuição da produção de lágrimas após iniciada após uso da medicação hipotensora, referida pela puérpera. Variável nominal, dicotômica do tipo sim/não.
- Tosse seca – presença de tosse sem expectoração, referida pela puérpera. Variável nominal, dicotômica do tipo sim/não.

- Erupções cutâneas – presença de lesões cutâneas que podem ser eritematosas ou ezematosas, referida pela paciente. Variável nominal, dicotômica do tipo sim/não.
- Prurido – coceira referida pela paciente. Variável nominal, dicotômica do tipo sim/não.
- Febre – sensação térmica elevada, medida por um termômetro que deve estar acima de 37,5ºC, referida ou diagnosticada por profissionais da área de saúde. Variável nominal, dicotômica do tipo sim/não.
- Artralgia – dor nas articulações referida pela puérpera. Variável nominal, dicotômica do tipo sim/não.
- Reações de fotossensibilidade – dificuldade em exrgar em ambientes claros referida pela puérpera. Variável nominal, dicotômica do tipo sim/não.
- Rubor – sinal eritematoso em pele referida pela puérpera. Variável nominal, dicotômica do tipo sim/não.
- Palidez – hipocromia corporal referida pela puérpera. Variável nominal, dicotômica do tipo sim/não.
- Hipotensão – queda dos níveis pressóricos com sintomas resentes, medida pelos profissionais de saúde utilizando tensiômetro e estetoscópio devidamente calibrados. Variável nominal, dicotômica do tipo sim/não.
- Taquicardia – frequência cardíaca acima de 100 batimentos, diagnosticado pelos profissionais de saúde através de monitor multiparâmetro ou atrvés do exame físico. Variável nominal, dicotômica do tipo sim/não.
- Dores no peito – dores na região torácica referida pela puérpera. Variável nominal, dicotômica do tipo sim/não.
- Palpitações – arritmia referida pela puérpera. Variável nominal, dicotômica do tipo sim/não.

- Disgeusia – sensaão de ausência ou diminuição do paladar referida pela puérpera. Variável nominal, dicotômica do tipo sim/não.
- Anemia – diminuição dos níveis do eritrograma (hemoglobina e hematócrito abaixo de 12g/dl e 30% respectivamente). Variável nominal, dicotômica do tipo sim/não.
- Trombocitopenia – diminuição do número de plaquetas abaixo de 150.000/mm$^{3.}$ Variável nominal, dicotômica do tipo sim/não.
- Insuficiência renal – alteração da função que pode ter origem pré, intra e pós rim. Variável nominal, dicotômica do tipo sim/não.
- Associação com outro agente hipotensor – necessidade de utilização de outra droga hipotensora, em decorrência da falta de controle dos níveis hipertensivos. Variável nominal, dicotômica do tipo sim/não.
- Tempo de internamento em UTI obstétrica – Variável numérica, discreta, expressa pelo número inteiro dos dias de internamento da puérpera em UTI obstétrica do IMIP.
- Frequência de picos hipertensivos – correspondendo ao número de picos hipertensivos (PAD $\geq$ 110mmHg e/ou PAS $\geq$ 170mmHg) ocorridos durante o acompanhamento da puérpera da admissão a alta da UTI obstétrica do IMIP. Variável numérica, discreta.
- Complicações maternas – variável categórica, tipo sim/não correspondendo às complicações maternas que vierem a ocorrer durante o estudo, como eclâmpsia, iminência de eclâmpsia, síndrome HELLP, pressão arterial não controlada e óbito materno. Variável nominal dicotômica do tipo sim/não.
- Oligúria – presença de fluxo urinário menor que 25 ml/h. Variável nominal, dicotômica do tipo sim/não.

- Óbito materno – como sendo a morte de uma mulher durante a gestação ou dentro de um período de 42 dias após o parto, independentemente da duração ou localização da gravidez, devido a qualquer causa relacionada com ou agravada pela gestação ou por medidas tomadas em relação a ela, porém não devidas a causas acidentais ou incidentais.[43] Entretanto, para esse estudo consideraremos apenas os óbitos materno devido ao aumento da pressão arterial na gravidez ou as medidas tomadas em relação a hipertensão. Variável nominal, dicotômica do tipo sim/não.
- Piora do quadro clínico materno – puérperas que no período puerperal evoluíram para um quadro clínico de maior gravidade, como eclâmpsia, iminência de eclâmpsia ou síndrome HELLP. Além, da evolução para complicações realicionadas a pré-eclâmpsia, como edema agudo de pulmão, coma, insuficiência respiratória, coagulação intravascular disseminada e insuficiência renal aguda. Variável nominal, dicotômica, tipo sim/não.

5.10. Procedimentos para Randomização, Seleção e Acompanhamento dos Sujeitos

5.10.1. Procedimentos para Randomização dos Sujeitos

A randomização para o tratamento hipotensor do pico pressórico, em puérperas com síndromes hipertensivas da gestação, será realizada de acordo com uma lista de números sequênciais de um a 70 (número de puérperas a serem randomizadas), pelo estatístico responsável utilizando o programa ”Random Allocation Software”, versão 1.0, empregando as letras A e B, sem conhecer o seu significado.

Por sorteio, o farmacêutico definirá, sem que os pesquisadores e o estatístico tenham acesso à informação, o significado das letras A e B, para captopril ou clonidina. O farmacêutico responsável pelo preparo das medicações e de posse dessas informações

deverá, por sua vez, colocar o captopril ou a clonidina em caixas padronizadas, segundo a randomização, mantendo essa informação em sigilo até o término da análise de dados.

As caixas serão preparadas de forma indistinguível, numeradas sequencialmente de um a 70, de acordo com a tabela de randomização. Cada caixa corresponderá a um conjunto de 10 comprimidos de captopril na dose de 25mg cada ou 10 comprimidos de clonidina na dose de 0,1mg cada. Prevendo-se a possibilidade de perda, intolerância e/ou vômitos durante a administração das medicações, a quantidade de comprimidos será superior à dose preconizada diária de cada droga (captopril, 150mg/dia e clonidina 0,6mg/dia), porém será respeitada sua utilização conforme protocolo de administração.[35] Garantir-se-á, portanto, tanto a ocultação da alocação como o triplo-mascaramento, uma vez que nem os pesquisadores, nem as puérperas e nem o estatístico terão conhecimento do conteúdo de cada caixa.

5.10.2. Procedimentos para preparo e administração das medicações

As caixas contendo 10 comprimidos de captopril na dose de 25mg ou 10 comprimidos de clonidina na dose de 0,1mg serão preparadas por um laboratório farmacêutico e serão adquiridas mediante pagamento após a aprovação deste projeto de pesquisa pela banca de qualificação do doutorado em Saúde Materno Infantil do IMIP e pelo Comitê de Ética em Pesquisa do IMIP. O significado de cada letra (A ou B), ou seja, do conteúdo de cada caixa, será conhecido exclusivamente pelo farmacêutico responsável. As caixas serão armazenadas na UTI obstétrica do IMIP, em local adequado, sob responsabilidade do pesquisador e das enfermeiras de plantão, para uso exclusivo nesta pesquisa.

Após inclusão no estudo, a caixa será entregue à técnica de enfermagem responsável pelo leito, explicando a forma de administração

por via oral sempre que a puérpera apresentar pico hipertensivo (PAS ≥ 170mmHg e/ou PAD ≥ 110mmHg), com intervalo de 20 minutos após cada aferição. A dose total de captopril não deverá exceder 150mg/dia, enquanto que a dose de clonidina não deverá ultrapassar 0,6mg/dia.

As puérperas ficarão cientes de seu quadro clínico, como também do conceito de pico hipertensivo. Entretanto, a responsabilidade sobre a administração da droga (captopril ou clonidina) será do técnico de enfermagem do setor.

5.10.3. Procedimentos para Seleção dos Sujeitos

Como forma de sensibilização de todos os médicos da UTI obstétrica do IMIP, relizar-se-á divulgação do projeto pelo pesquisador responsável antes de iniciada a coleta de dados, por meio de cartazes e aviso por escrito (Apêndice 1)

O médico plantonista e/ou médico residente da UTI obstétrica do IMIP identificará as possíveis puérperas, com síndromes hipertensivas, candidatas ao estudo, de agosto de 2012 a novembro de 2012. Após terem sido admitidas neste setor, deve ser iniciada, conforme rotina, a administração da dose de ataque – seis gramas por via intravenosa – do sulfato de magnésio, em 20 minutos, quando necessário, além da manutenção por 24 horas pós-parto (Figura 1).

Em uso do sulfato de magnésio, serão verificadas a frequência cardíaca e pressão arterial (PAS, PAD, PAM), repetindo-se o exame de proteinúria de fita em até duas horas de admissão da puérpera na UTI obstétrica (Figura 1). Após confirmação do pico hipertensivo, aplicar-se-á a lista de checagem, contendo os critérios de inclusão e

exclusão do estudo (Apêndice 2). Se a puérpera for considerada elegível, receberá explicações sobre o motivo da pesquisa e sua importância (Figura 1).

Todas as puérperas incluídas no estudo serão devidamente esclarecidas sobre os objetivos e as possíveis consequências de sua participação. As puérperas serão ainda informadas que caso não aceitem mais participar, o seu acompanhamento será realizado segundo a rotina habitual do serviço por equipe capacitada (Figura 1).

Caso concordem voluntariamente em participar, será solicitada a sua assinatura no Termo de Consentimento Livre e Esclarecido (Apêndice 3), após leitura cuidadosa e explicações pelo pesquisador.

Os pesquisadores responsáveis, diariamente, no período matinal, irão se deslocar ao setor de pré-parto e UTI obstétrica identificando as puérperas que já foram incluídas ou excluídas do estudo (pelos médicos plantonistas) e aquelas que poderão ainda ser candidatas à inclusão (Figura 1).

5.10.4. Acompanhamento dos Sujeitos

Após a inclusão no estudo, será iniciada a administração da medicação hipotensora para o pico pressórico (captopril ou clonidina), de acordo com a randomização, conforme já descrito na seqüência de procedimentos (Figura 1). A puérpera receberá um número ordinal, sequencial, correspondendo a uma caixa lacrada, contendo 10 comprimidos de captopril 25mg cada, ou 10 comprimidos de clonidina 0,1mg cada. Todos os prontuários serão devidamente identificados com o mesmo número seqüencial da puérpera.

As caixas contendo os comprimidos serão padronizadas. Cada caixa, além do número sequencial, será identificada com o nome e o registro da puérpera, e somente

será aberta em sua frente, sob responsabilidade do técnico de enfermagem e da enfermeira do setor.

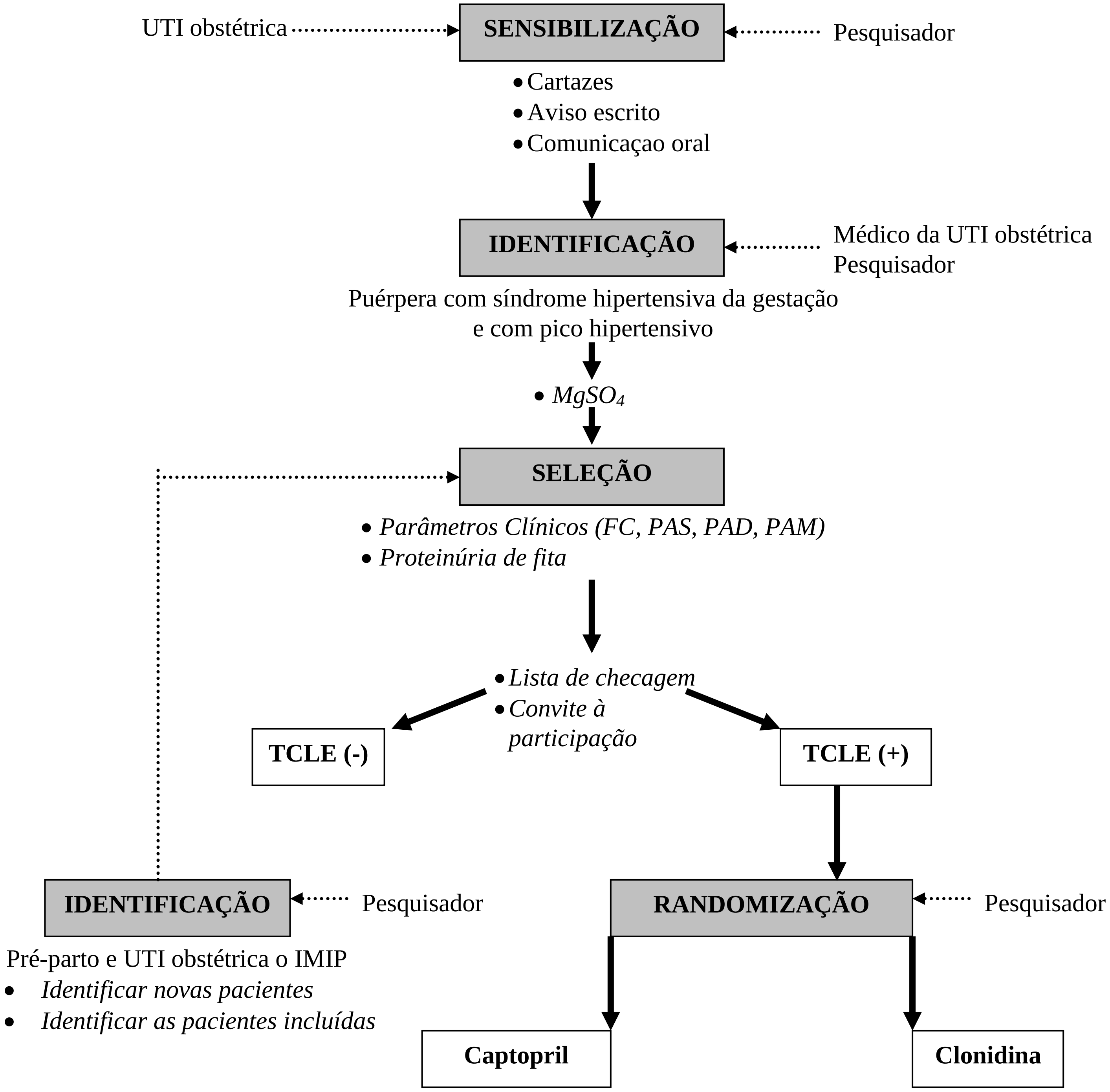

Figura 1 – Procedimentos para seleção e acompanhamento dos sujeitos.

- TCLE: termo de consentimento livre e esclarecido; PAS: pressão arterial sistólica; PAD: pressão arterial diastólica; PAM: pressão arterial média; FC: frequência cardíaca, $MgSO_4$: sulfato de magnésio.

Em nenhum momento, pesquisadores, puérperas e estatístico terão conhecimento de qual medicação (captopril ou clonidina) está sendo administrada durante o acompanhamento obstétrico, até o término da coleta de dados e análise. Desta forma, será garantida a ocultação da alocação. Somente o farmacêutico, responsável pela pesquisa, terá conhecimento do conteúdo das caixas através de randomização estabelecida pelo estatístico responsável, sendo revelada apenas após a análise dos dados.

Um fluxograma será preenchido, respeitando as recomendações do *Consort Statement*,[48] conforme mostrado a seguir (Figura 2), com o perfil do ensaio clínico, incluindo o número total de puérperas, admitidas na UTI obstétrica do IMIP, com diagnóstico de síndromes hipertensivas da gestação e dessas quantas foram consideradas com pico hipertensivo no período do estudo, o número de puérperas elegíveis, o número daquelas a quem foi oferecido participar do estudo, o número de recusas, o número das puérperas que aceitaram participar, o número de perdas durante o estudo, o número de puérperas excluídas durante o estudo e o número alocado para receber captopril ou clonidina. Esse último somente será conhecido após a análise estatística.

Todos os prontuários das puérperas envolvidas no estudo serão identificados através de etiquetas autocolantes contendo o nome da pesquisa e seu número de identificação, correspondente ao número da caixa que lhe for destinada, para revisões posteriores que se façam necessárias. Além disso, todas as puérperas receberão um cartão contendo um número de telefone celular (81-9234-0457), destinado especificamente para a pesquisa, para contato com o pesquisador, caso necessário, podendo a ligação ser feita a cobrar.

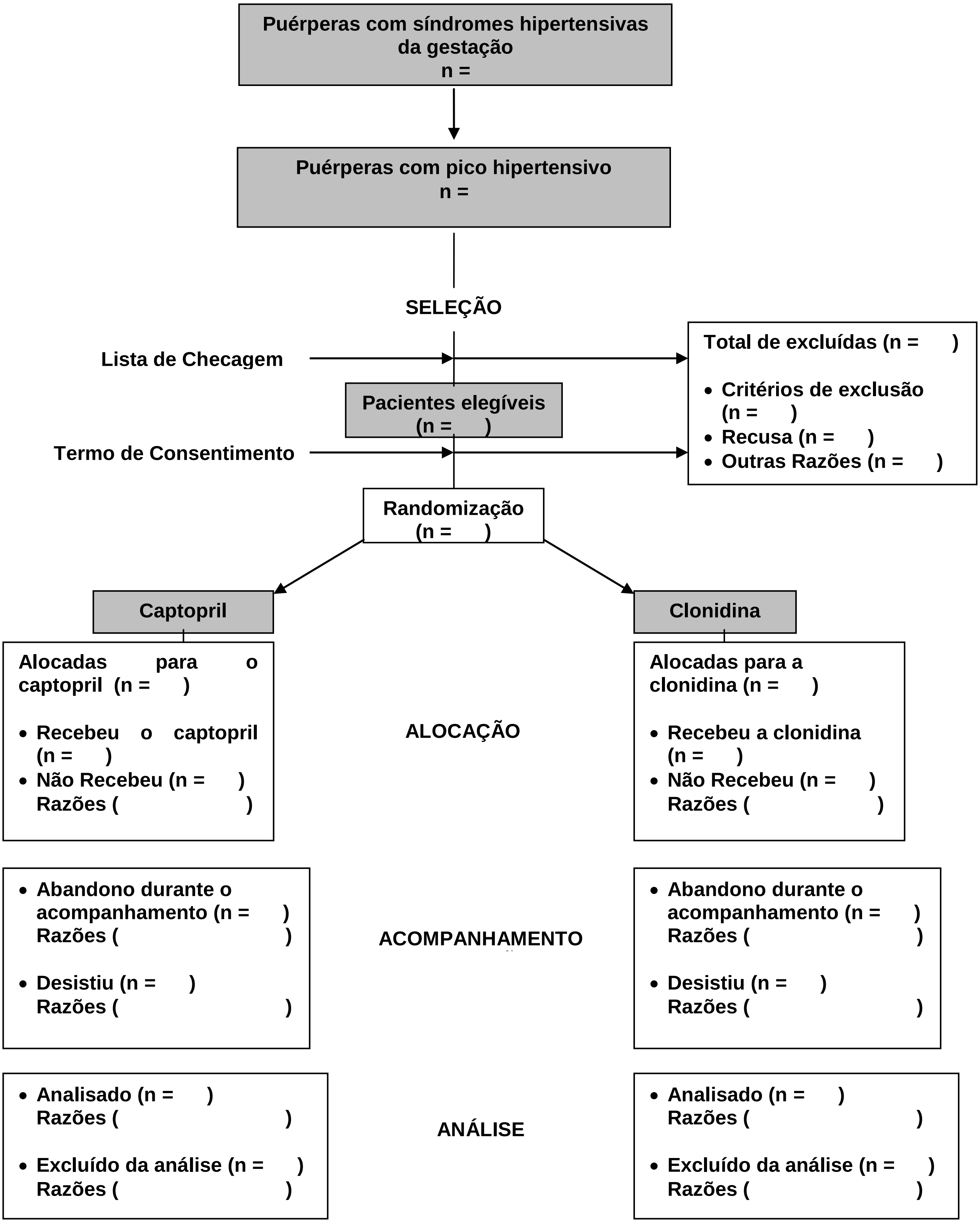
Puérperas com síndromes hipertensivas da gestação
n =
Puérperas com pico hipertensivo
n =
SELEÇÃO
Lista de Checagem
Total de excluídas (n =)
Critérios de exclusão (n =)
Recusa (n =)
Outras Razões (n =)
Pacientes elegíveis (n =)
Termo de Consentimento
Randomização (n =)
Captopril
Clonidina
Alocadas para o captopril (n =)
Recebeu o captopril (n =)
Não Recebeu (n =)
Razões ( )
ALOCAÇÃO
Alocadas para a clonidina (n =)
Recebeu a clonidina (n =)
Não Recebeu (n =)
Razões ( )
Abandono durante o acompanhamento (n =)
Razões ( )
Desistiu (n =)
Razões ( )
ACOMPANHAMENTO
Abandono durante o acompanhamento (n =)
Razões ( )
Desistiu (n =)
Razões ( )
Analisado (n =)
Razões ( )
Excluído da análise (n =)
Razões ( )
ANÁLISE
Analisado (n =)
Razões ( )
Excluído da análise (n =)
Razões ( )

Figura 2 – Procedimentos para seleção e acompanhamento dos sujeitos (Fluxograma CONSORT).[48]

5.11. Caracterização Amostral

As puérperas, admitidas no estudo, serão avaliadas de acordo com suas diversas características, levando em consideração a formação dos dois grupos (captopril e clonidina), com o objetivo de testar o processo de randomização e a comparabilidade entre os grupos.

5.12. Procedimentos, Testes, Técnicas e Exames

- **Procedimentos para aferição da pressão arterial** – No momento da admissão em UTI obstétrica do IMIP, para aferição da pressão arterial da puérpera, será utilizado um monitor multiparamétrico modelo Dixtal devidamente calibrado para suas funções propostas. As pacientes estarão em uso de sulfato de magnésio, deitadas no respectivo leito, com esfingmomanometro do monitor multiparamétrico afixado em seu membro superior direito. Após confirmação do aumento dos níveis hipertensivos (PAS $\geq$ 170mmHg e/ou PAD $\geq$ 110 mmHg) a puérpera será elegível para o estudo. Caso contrário, a puérpera permanecerá na UTI obstétrica do IMIP, sendo incluída no estudo apenas se, posteriormente, preencher os critérios de inclusão. A pressão arterial será aferida durante o internamento hospitalar segundo a rotina do serviço, ou seja, a cada duas horas no período de administração do sulfato de magnésio e a cada quatro horas diariamente, pelo técnico de enfermagem do setor. Todas as aferições serão realizadas com o monitor multiparamétrico. Para a análise estatatística serão consideradas todas as aferições de pressões arteriais.

- **Procedimento para determinação da proteinúria de fita** – será medida na admissão da puérpera na UTI obstétrica do IMIP, em duas amostras de urina, coletadas com intervalo de 30 minutos pelo médico assistente através de fita de Labstix®. Posteriormente, poderá ser realizada durante o internamento no setor. O valor qualitativo será expresso em uma escala variando de negativo, traços e uma a quatro cruzes (+). A fita ficará acondicionada em local e temperatura adequados, seguindo rigorosamente as normas do fabricante. Essas fitas não serão utilizadas após a data de expiração, serão guardadas em frasco original, não sendo expostas à luz e nem umidade, os frascos deverão encontrar-se bem fechados e as fitas serão retiradas apenas quando forem ser utilizadas.
- **Procedimentos para realização dos exames laboratoriais** – todos os exames laboratoriais serão realizados inicialmente no momento da admissão da puérpera na UTI obstétrica, segundo a rotina do serviço. Portanto, a intervenção (captopril ou clonidina) será iniciada apenas após a coleta dos exames laboratoriais. Posteriormente, todos os exames (plaquetas, uréia, creatinina, ácido úrico, AST, ALT, DHL, sódio, potássio e cloro) serão realizados diariamente, segundo as técnicas habituais do laboratório de hematologia e bioquímica do IMIP.
- **Acompanhamento da puérpera durante o internamento na UTI obstétrica** – durante o internamento hospitalar da puérpera para controle dos níveis hipertensivos, o acompanhamento será realizado de acordo com a rotina do serviço, conforme preconizado no Manual de Normas da Instituição.[43] Assim, os níveis hipertensivos serão aferidos diariamente, pelos técnicos de enfermagem, a cada duas horas, na vigência de $MgSO_4$ e a cada quatro horas após suspensa a droga anticonvulisvante. Os exames laboratoriais serão realizados diariamente, nos

primeiros dias, até que se tenham duas rotinas normais, quando então as solicitações para acompanhamento laboratorial passam a ser desnecesárias. (Figura 3).

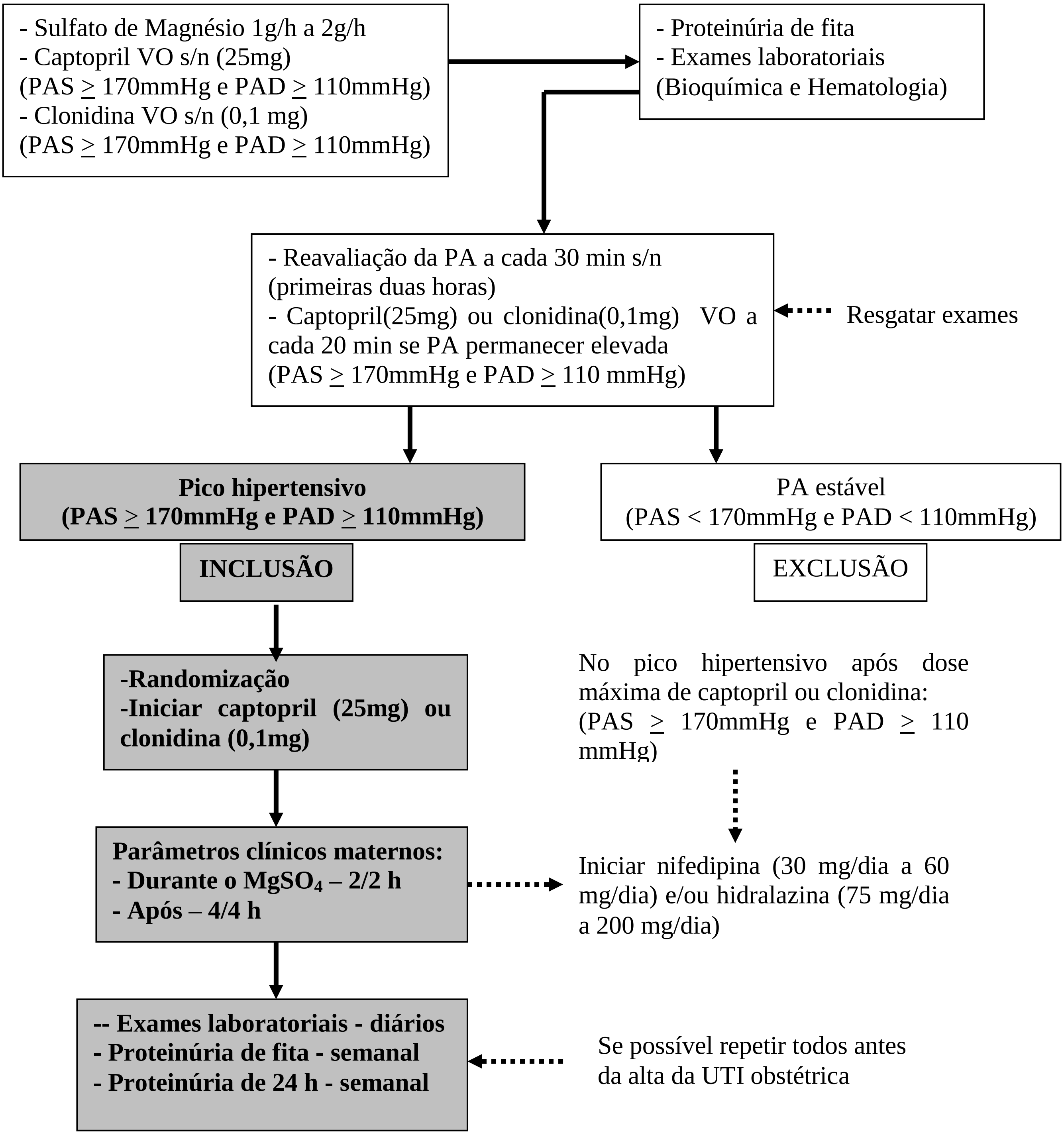

Figura 3 – Fluxograma acompanhamento das puérperas.

- **Terapêutica com o sulfato de magnésio pós-parto** – será instituída, ainda no setor de pré-parto, em todas as puérperas com pré-eclâmpsia grave ou hipertensão crônica agravada pela gestação. Será adotado o esquema de Zuspan modificado,[49] conforme preconizado no Manual de Normas da Instituição,[43,50] correspondendo a seis gramas intravenosa (dose de ataque) e a seguir infusão de um grama por hora (manutenção). Será utilizada a apresentação do sulfato de magnésio a 10%, cada frasco-ampola contendo 10ml da solução. Para a realização do ataque será preparada uma solução com 40ml de soro glicosado a 5% e 60ml de sulfato de magnésio a 10%, que correrá em bomba de infusão por 20 minutos. Para a realização da manutenção será preparada uma solução com 440ml de soro glicosado a 5% e 60ml de sulfato de magnésio a 10%, programada para correr em bomba de infusão em seis horas (84ml/h). A puérpera será reavaliada a cada seis horas pelo médico plantonista. Serão considerados os seguintes parâmetros clínicos: frequência respiratória de pelo menos 14 incursões por minuto, reflexos patelares presentes bilateralmente e normoativos e diurese superior a 25 ml/h.[3,50] Caso seja encontrado algum desses parâmetros alterados, a manutenção do sulfato de magnésio será suspensa até a normalização do quadro clínico.
- **Critérios para avaliar a necessidade de medicação no pico hipertensivo** – durante o internamento da puérpera, caso seja diagnosticado pico hipertensivo (PAS $\geq$ 170mmHg e/ou PAD $\geq$ 110mmHg), será instituído o tratamento. A medicação em estudo será o captopril oral, 25mg, a cada 20 minutos (dose total de 150mg) ou a clonidina oral, 0,1mg a cada 20 minutos (dose total de 0,6mg) até queda da pressão arterial a níveis abaixo da urgência hipertensiva.
- **Critérios para avaliar a necessidade de associação com outra droga** – nos picos hipertensivos, o captopril ou a clonidina serão administrados na dose de 25mg e

0,1mg, respectivamente. Caso não haja controle da pressão arterial em 20 minutos, novas doses de hipotensor serão administradas até um total de 150mg/dia (seis comprimidos) de captopril e 0,6mg/dia (seis comprimidos) de clonidina. Após ultrapassado a dose permitida, outra droga poderá ser associada. Inicialmente, a nifedipina (30mg/dia a 60mg/dia) de acordo com a rotina do serviço. O objetivo da intervenção é o de manter a pressão arterial sistólica abaixo de 170mmHg e a diastólica inferior a 110mmHg, com a menor dose possível. Assim, essas medicações serão aumentadas, caso haja necessidade, de acordo com as aferições realizadas diariamente pelo médico assistente e pelos técnicos de enfermagem.

- **Critérios de alta da UTI obstétrica do IMIP** – as puérperas que evoluírem com boas condições clínicas, exames laboratoriais normais e sem complicações pós-parto receberão alta do setor, pelo médico assistente, após controle dos níveis pressóricos e serão acompanhadas no ambulatório da mulher do CAM-IMIP pelo médico assistente responsável pelo retorno das puérperas e pelo cardiologista.
- **Acompanhamento ambulatorial** – será realizado acompanhamento ambulatorial com retornos na sexta e 12ª semana pós-parto e a seguir semestralmente durante dois anos, registrando-se a pressão arterial e a eventual necessidade de tratamento hipotensor.

5.13. Procedimentos para Coleta de Dados

5.13.1. Instrumento de Coleta

Os dados serão coletados pelo próprio pesquisador, utilizando um formulário padronizado, com perguntas fechadas e pré-codificadas para entrada no computador (Apêndice 4). Esse instrumento será testado em um estudo piloto que sofrerá as modificações necessárias para se adequar aos objetivos da pesquisa.

As informações das variáveis categóricas serão pré-codificadas e as variáveis contínuas expressas em seu próprio valor numérico e só no momento da análise dos resultados, algumas poderão ser, então, categorizadas.

Esses formulários serão devidamente armazenados em pastas de arquivo específicas, antes e depois da digitação e análise, sob responsabilidade do próprio pesquisador, que irá preenchê-los durante o período pós parto.

5.13.2. Coleta de Dados

Os dados serão coletados pelo próprio pesquisador e um colaborador, que poderá ser um médico residente em Tocoginecologia ou um aluno de medicina da graduação, devidamente treinado (bolsista de Iniciação Científica). Esses aplicarão a lista de checagem (Apêndice 2) nas puérperas candidatas ao estudo, para incluí-las na pesquisa. Posteriormente, aplicar-se-á o protocolo do estudo e os formulários (Apêndice 4) serão preenchidos, registrando os resultados da avaliação clínica e obstétrica da puérpera.

Após preenchimento, os formulários serão revisados rigorosamente pelo pesquisador para a checagem das informações coletadas com as informações constantes nos prontuários. O tempo transcorrido para coleta de dados, preenchimento adequado de formulários e sua revisão deverá corresponder ao previsto no cronograma.

5.13.3. Controle de Qualidade das Informações

Os formulários serão preenchidos diariamente pelos pesquisadores e arquivados em pasta específica para a pesquisa, no Departamento de Pesquisa do IMIP. Serão revisados, em blocos de dez, pelos próprios pesquisadores. Em se constatando ausência ou incorreção de informações, serão consultados os prontuários e os resultados dos exames laboratoriais para elucidação das dúvidas.

5.14. Critérios para Descontinuação do Estudo

Critérios para descontinuação individual (exclusão de uma puérpera do estudo):

- ❖ Recusa da puérpera em prosseguir com os procedimentos, mesmo depois de assinado o termo de consentimento;
- ❖ Complicações puerperais relacionadas ou não às síndromes hipertensivas e doenças maternas diagnosticadas durante o estudo;
- ❖ Reações graves inesperadas.

Critérios para descontinuação do estudo (término do recrutamento de puérperas):

- ❖ Reações graves inesperadas: algum efeito ligado as drogas captopril/clonidina ameaçando a vida das puérperas ou levando a incapacidade física ou psíquica (extremamente improvável porquanto nunca foram descritos na literatura);

5.15. Comitê de Monitoramento Externo (CME)

O comitê de monitoramento dos dados será um grupo de pesquisadores independentes e externos ao estudo que assegurará a progressão e segurança dos dados e, se necessário, criticará a efetividade e poderá finalizar o ensaio clínico. Apenas o

CME poderá dispor de informações quando necessário sobre o cegamento do ensaio clínico.

5.15.1. Membros do CME e suas qualificações

- Ana Maria Feitosa Porto: ginecologista e obstetra do IMIP.
- Alex Sandro Rolland de Souza: ginecologista e obstetra do IMIP.
- José Natal Figueroa: estatístico da pós-graduação do IMIP. .
- Aurélio Ribeiro da Costa: ginecologista e obstetra do IMIP.
- Adriana Scavuzzi: ginecologista e obstetra do IMIP.

5.15.2. Responsabilidades dos Membros do CME

- Ana Porto: coordenadora do grupo. Responsável pela marcação das reuniões semestrais do grupo e coordenar as discussões das reuniões.
- Alex Sandro Rolland de Souza: secretário. Responsável por registrar em ata as discussões das reuniões e suas decisões. Além de solicitar os formulários de coleta e/ou banco de dados ao pesquisador responsável.
- José Natal Figueroa: estatístico e aspectos éticos. Responsável pela análise de ínterim quando pertinente e os aspectos éticos envolvidos no estudo.
- Aurélio Ribeiro da Costa: avaliador. Responsável pelas discussões do grupo e pelo relatório final.
- Adriana Scavuzzi: avaliadora. Responsável pelas discussões do grupo e pelo relatório final.

5.15.3. Conflitos de Interesse dos Membros do CME

- Ana Maria Feitosa Porto: não há.

- Alex Sandro Rolland de Souza : não há.
- José Natal Figueroa: ao final do estudo o mesmo poderá orientar nas análises estatísticas, se necessário.
- Aurélio Ribeiro da Costa: não há.
- Adriana Scavuzzi: não há.

5.15.4. Frequência e formato das reuniões do CME

As reuniões serão mensais em sala reservada na pós-graduação do IMIP, sem o conhecimento dos pesquisadores envolvidos diretamente com o estudo.

Inicialmente será realizada uma análise da frequência dos principais desfechos estudados, comparando-se com a frequência desses achados em estudos observacionais já realizados no IMIP e/ou dispostos na literatura. Esses estudos serão disponibilizados para os membros do CME. Essa análise será realizada sem a quebra do sigilo e sem identificação do grupo de tratamento. Em se constatando frequências dos eventos desfavoráveis maiores que o esperado para a amostra estudada, ou se ocorrerem eventos inesperados, o CME deverá sugerir a realização de analise de ínterim.

5.15.5. Fluxo das reuniões do CME

As reuniões serão marcadas pela coordenadora do comitê de monitoramento externo, confirmando a presença de todos os membros. O secretário solicitará ao pesquisador responsável o material da pesquisa colhido no período anterior à reunião. Na reunião, todos os membros irão calcular as frequências dos principais defechos listados no item 5.15.6. Caso seja evidenciado alguma frequência do desfecho acima do esperado (baseado na literatura), o CME decidirá sobre a necessidade da análise de

ínterim. O estatístico ficará responsável por essa análise, sendo nova reunião marcada para decisão final sobre a continuação da pesquisa (Figura 4).

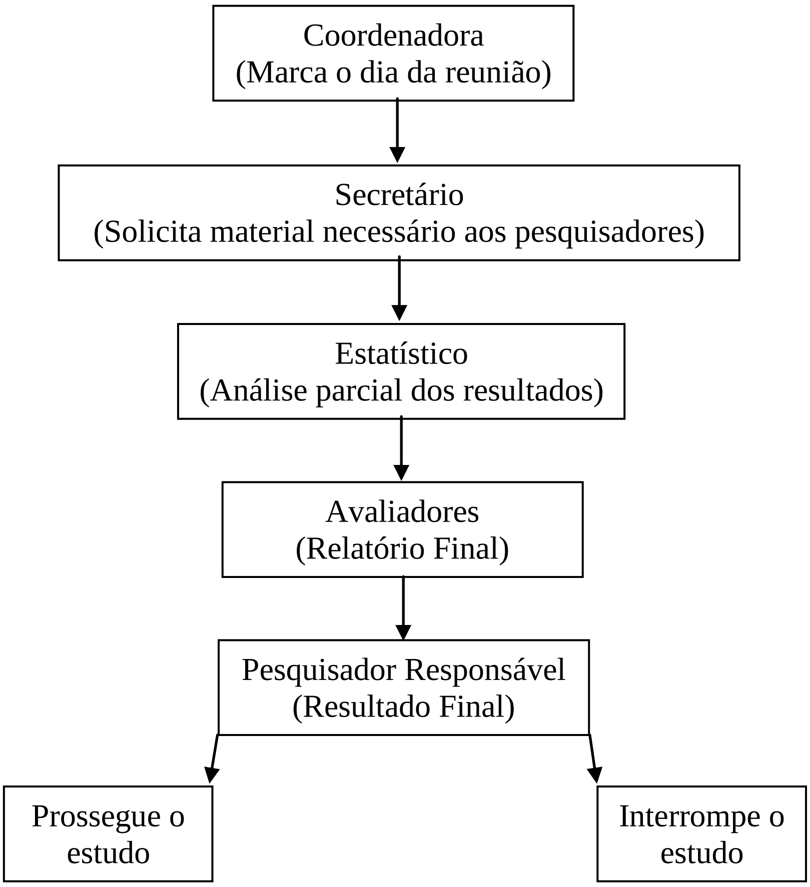

Figura 4 – Fluxo das reuniões do CME.

5.15.6. Eventos adversos monitorados

Os eventos adversos ao uso de clonidina[38] e do captopril[32] serão monitorados a cada mês observando se a frequência encontra-se maior do que é previsto na literatura e em estudos anteriores realizados no IMIP:

- Boca seca e sedação (50%);
- Sonolência (35%);
- Tontura (35%);
- Hipotensão arterial (5%)
- Tosse seca (2%);
- Prurido (2%);
- Artralgia (4%);

5.15.7. Plano de análise de ínterim

A análise de ínterim será realizada se alguns dos desfechos apresentarem uma frequência acima do esperado para a amostra estudada, devendo-se quebrar o sigilo para averiguar se um dos grupos está acarretando maiores riscos às participantes e/ou seus conceptos.

5.16. Processamento e Análise dos Dados

5.16.1. Processamento dos Dados

Os dados serão digitados em banco de dados específico criado no programa estatístico de domínio público Epi-Info 7 para a versão Windows Vista. A digitação dos dados será realizada após revisão dos formulários. Mensalmente esse banco de dados será revisado pelo pesquisador, obtendo listagem das variáveis e corrigindo eventuais inconsistências ou falta de informações a partir da consulta aos formulários.

Será realizada dupla digitação, em épocas e por pessoas diferentes (o pesquisador e um assistente). Após a digitação, os bancos de dados serão comparados. Testes de consistência serão adotados e tabelas de distribuição de frequências das principais variáveis serão preparadas para correção de eventuais erros. No caso da

constatação de inconsistências ou ausência de informações, os formulários serão consultados. Persistindo a dúvida, recorrer-se-á aos prontuários.

Ao final da digitação, serão novamente obtidas listagens, para a correção final e criação do banco de dados definitivo, que será submetido aos testes de limpeza e consitência das informações e a partir daí será realizada a análise estatística.

5.16.2. Análise de Dados

A análise estatística será realizada pelo pesquisador e seus orientadores, além do estatístico envolvido na pesquisa, utilizando os programas estatísticos Epi-Info 7 e Medcalc software 12.2.1.0 para Windows.

A análise estatística será realizada com os grupos identificados como A ou B, quebrando-se o sigilo somente depois de obtidos os resultados e preparadas tabelas. Assim, só se conhecerá qual grupo recebeu captopril ou clonidina ao final da análise.

As variáveis categóricas serão comparadas em tabelas de contingência utilizando-se os testes qui-quadrado de associação e exato de Fisher, quando pertinente. Será calculada a razão de risco (RR) como medida de risco relativo, determinando-se seu intervalo de confiança de 95%. Ainda serão calculados os números necessários para tratar e obter um benefício (NNT) e o número necessário para tratar e obter um malefício (NNH) e seus respectivos intervalos de confiança a 95%. Esse cálculo será realizado com a calculadora Evidence Based Calculator, disponível no site http://moosenose.com/EBCalculator.htm.

Em relação às variáveis quantitativas contínuas com variâncias dessemelhantes, caso apresentem distribuição normal, a comparação entre os grupos será realizada com o teste *t de Student* para amostras não pareadas (testes paramétricos). Caso se verifique que a distribuição não é normal, será utilizado o teste não paramétrico de *Mann-*

Whitney. Esses testes serão utilizados para identificar as diferenças pontuais entre os dois grupos. A análise de sobrevivência de Kaplan-Meyer será realizada com a intenção de verificar a evolução das medições de PAS, PAD e PAM nos dois grupos, calculando-se o *log rank.*

Todos os valores de p adotados serão bicaudados e em todas as etapas da análise se adotará o nível de significância de 5%.

5.17. Aspectos éticos

O presente estudo atende aos requisitos da "Declaração de Helsinque"[51] para pesquisa em seres humanos, e à resolução 196/96 do Conselho Nacional de Saúde.[52] O projeto será submetido à apreciação do Comitê de Ética em Pesquisa (CEP) do IMIP, tendo início somente após sua aprovação. Também será registrado na plataforma REBEC (Registro Brasileiro de Ensaios Clínicos) e no Clinical Trials.gov.

Todas as participantes serão devidamente esclarecidas sobre os objetivos do estudo e somente serão incluídas caso concordem em participar e assinem o "Termo de Consentimento Livre e Esclarecido" (Apêndice 3).

O principal objetivo deste estudo é definir qual a melhor droga hipotensora para os casos de puérperas com síndromes hipertensivas da gestação e picos hipertensivos. O captopril é um inibidor da enzima conversora de angiotensina que é atualmente empregado no tratamento dos picos hipertensivos nesse grupo particular de mulheres que sofrem as modificações do período pós-parto. A clonidina, empregada também em procedimentos anestésivos, traz benefícios hipotensores documentados em alguns relatos da literatura.

Há algum tempo, a clonidina começou a ser empregada na UTI obstétrica do IMIP com a intenção de tratar os picos hipertensivos em puérperas selecionadas que não

estavam aptas ao uso de captopril. Com o tempo, verificou-se que a clonidina poderia trazer resultados mais precoces e duradouros na manutenção da pressão arterial no puerpério.

Apesar de o estudo ser um ensaio clínico randomizado para a utilização da captopril ou clonidina e ter como principal objetivo avaliar o tratamento hipotensor dos picos hipertensivos nas puérperas com síndromes hipertensivas da gestação, as participantes incluídas não ficarão desprovidas de medicação hipotensora, caso haja necessidade. No grupo do captopril ou da clonidina, as puérperas realizarão a medicação de acordo com a aferição de PAS ≥ 170mmHg e/ou PAD ≥ 110mmHg por até cinco vezes com intervalo de 20 minutos. Em contrapartida, depois de atingida a dose total das drogas, outra medicação hipotensora poderá ser empregada como manutenção ou tratamento associado da pressão arterial instável. Ressalta-se que as aferições da pressão arterial serão realizadas regularmente a cada duas horas enquanto as puérperas estiverem em uso de sulfato de magnésio e a cada quatro horas após finalizada medicação anticonvulsivante. Desta forma, caso ocorram picos hipertensivos, as puérperas serão medicadas no intervalo mínimo de quatro horas.

Trata-se de um ensaio clínico randomizado, de curta duração, em que não será alterada a rotina do serviço. Em nosso estudo, serão avaliados os efeitos do tratamento hipotensor para os picos hipertensivos, utilizando o captopril e a clonidina nas puérperas com síndrome hipertensiva da gestação. Foram escolhidos vários parâmetros clínicos e obstétricos, como se descreve na seção de Métodos. A avaliação de todas as variáveis será realizada habitualmente como rotina da instituição para esse grupo específico de puérperas, o que não trará nenhum prejuízo para o IMIP ou mesmo para as puérperas. Todos os procedimentos, técnicas e exames descritos também já são realizados rotineiramente pelos profissionais da instituição.

O captopril e a clonidina são utilizados habitualmente no IMIP como drogas hipotensoras. Vários estudos sugerem que são seguras e eficazes para essa finalidade, não havendo descrição na literatura de complicações graves com seus usos.

Serão excluídas do estudo as puérperas que apresentarem, além dos distúrbios hipertensivos, complicações clínicas que possam piorar os níveis hipertensivos a tal ponto que contribuam para um maior risco de morte entre as participantes, como por exemplo: cardiopatias, colagenoses, diabetes Mellitus, pneumopatias e síndromes nefróticas.

Não existirá vinculação financeira ou empregatícia do pesquisador e seus orientadores com o laboratório que for escolhido para produzir as medicações (captopril e clonidina). O laboratório farmacêutico apenas preparará os medicamentos e não fornecerá subsídios ou bolsas para os pesquisadores.

O pesquisador e seus orientadores se comprometem a publicar os resultados do estudo em periódico indexado no MEDLINE, independente de favorecer essa ou aquela droga para o tratamento hipotensor do pico pressórico em puérperas com síndomes hipertensivas da gestação.

VI – RESULTADOS (*DUMMY TABLES*)

Os resultados serão apresentados em tabelas como se segue, ou em gráficos quando pertinentes:

Tabela 1 – Características basais das puérperas com picos hipertensivos, de acordo com o uso de captopril ou clonidina

Característica	Captopril	Clonidina	*p*
**Idade (anos)**			
X + DP			
**Paridade**			
Mediana – IIQ			
**Gestações**			
Mediana – IIQ			
**Id. Gestacional no parto (sem)**			
X + DP			
**Forma Clínica da Síndrome Hipertensiva (n, %)**			
Hipertensão Crônica			
Pré-eclâmpsia/ eclampsia			
Pré-eclâmpsia superposta			
Hipertensão gestacional			

X: média, IIQ: segundo quartil, DP: desvio padrão, Id.: idade, Sem: semanas, IIQ: intervalo interquartil, n: amostra, %: percentagem

p: teste estatístico

Tabela 2 – Frequência cardíaca e pressão arterial das puérperas com pico hipertensivo no momento da admissão, de acordo com o uso de captopril ou clonidina.

**Parâmetro clínico**	**Captopril**		**Clonidina**		***p***
	**Média ± DP**	**Variação**	**Média ± DP**	**Variação**	
**PAS (mmHg)**					
**PAD (mmHg)**					
**PAM (mmHg)**					
**FC (bpm)**					

PAS: pressão arterial sistólica, PAD: pressão arterial diastólica, PAM: pressão arterial média, FC: frequência cardíaca, mmHg: milímetro de mercúrio, bpm: batimentos por minuto, DP: desvio padrão, p: teste estatístico.

Tabela 3 – Exames laboratoriais das puérperas com pico hipertensivo no momento da admissão, de acordo com o uso de captopril ou clonidina.

	Captopril		Clonidina		
**Exames laboratoriais**	**Média + DP**	**Variação**	**Média + DP**	**Variação**	**p**
Plaquetas / mm^3					
Uréia (mg/dl)					
Creatinina (mg/dl)					
Ácido Úrico (mg/dl)					
DHL (U/l)					
AST (U/l)					
ALT (U/l)					
Bilirrubinas totais (mg/dl)					
Bilirrubina ind. (mg/dl)					
Bilirrubina direta (mg/dl)					
Proteinúria de fita					
Sódio (mEq/l)					
Potássio (mEq/l)					
Cloro (mEq/l)					

DP: desvio padrão, p: teste estatístico, mm^3: milímetro cúbico, mg/dl; miligrama por decilitro, U/l: unidades por litro, DHL: desidrogenase láctica, AST: aspartato transferase, ALT: alanina transferase, mEq/l: miliequivalentes por litros, ind: indireta, p: teste estatístico.

Tabela 4 – Exames laboratoriais nas puérperas com pico hipertensivo durante o internamento na UTI obstétrica, de acordo com o uso de captopril ou clonidina.

Exames laboratoriais	Captopril		Clonidina		p
	**Média ± DP**	**Variação**	**Média ± DP**	**Variação**	
Plaquetas / mm^3					
Uréia (mg/dl)					
Creatinina (mg/dl)					
Ac. Úrico (mg/dl)					
DHL (U/l)					
AST (U/l)					
ALT (U/l)					
Bilirrubinas totais (mg/dl)					
Bilirrubina ind. (mg/dl)					
Bilirrubina direta (mg/dl)					
Proteinúria de fita					
Sódio (mEq/l)					
Potássio (mEq/l)					
Cloro (mEq/l)					

DP: desvio padrão, p: teste estatístico, mm^3: milímetro cúbico, mg/dl; miligrama por decilitro, U/l: unidades por litro, DHL: desidrogenase láctica, AST: aspartato transferase, ALT: alanina transferase, mEq/l: miliequivalentes por litro, ind: indireta

.

Tabela 5 – Efeitos adversos em puérperas com pico hipertensivo durante o internamento na UTI obstétrica, de acordo com o uso de captopril ou clonidina.

**Efeitos adversos**	**Captopril**		**Clonidina**		
	**N**	**%**	**N**	**%**	***p***
Secura da boca					
Sensação de cansaço					
Distúrbio ortostático					
Vertigem					
Obstipação					
Transtornos do sono					
Distúrbios circulatórios					
Transtornos da percepção					
Pesadelo					
Dores das glândulas parótidas					
Estado de depressão					
Secura da mucosa nasal					
Diminuição da lacrimação					
Secura da boca					
Tosse seca					
Erupções cutâneas					
Prurido					

Febre
Artralgia
Lesão de pele tipo penfigóide
Reações de fotossensibilidade
Rubor
Palidez
Hipotensão
Taquicardia
Dores no peito
Palpitações
Disgeusia
Anemia
Trombocitopenia
Pancitopenia
Angioedema
Insuficiência renal

N: amostra, %: percentagem, p: teste estatístico

Tabela 6 – Médias do tempo de internamento em UTI obstétrica e idade gestacional do parto nas puérperas com pico hipertensivo durante o internamento na UTI obstétrica, de acordo com o uso de captopril ou clonidina.

**Característica**	**Captopril**		**Clonidina**		
	**Média ± DP**	**Variação**	**Média ± DP**	**Variação**	***p***
**Tempo de internamento em UTI obstétrica do IMIP (dias)**					
**IG. parto (sem)**					

DP: desvio padrão, p: teste estatístico, IG: idade gestacional, UTI: unidade de terapia intensiva, IMIP: Instituto de Medicina Integral professor Fernando Figueira, Sem: semana

Tabela 7 – Avaliação da efetividade da clonidina nas puérperas com pico hipertensivo durante o internamento na UTI obstétrica, de acordo com o uso de captopril ou clonidina.

Desfecho	Captopril		Clonidina		*p*
	**N**	**%**	**N**	**%**	
*** Necessidade de outro agente hipotensor**					
*** Necessidade de suspender o Tratamento**					
*** Motivo de suspender o Tratamento**					
Efeitos adversos					
Outros					

N: amostra, %: percentagem, p: teste estatístico

Tabela 8 – Complicações maternas em puérperas com pico hipertensivo durante o internamento na UTI obstétrica, de acordo com o uso de captopril ou clonidina.

Complicações	Captopril		Clonidina		RR	IC	p
	N	%	N	%			
**DPPNI**							
**Eclâmpsia**							
**Síndrome HELLP**							
**Iminência de eclampsia**							
**Óbito materno**							

N: amostra, %: percentagem, p: teste estatístico, DPPNI: descolamento prematuro de placenta normalmente inserida, HELLP: hemolises, low plaquet, elevator liver, RR: risco relativo, IC: intervalo de confiança, p: teste estatístico

Tabela 9 – Pressão arterial e frequência cardíaca materna em puérperas com pico hipertensivo durante o internamento na UTI obstétrica, de acordo com o uso de captopril ou clonidina.

Parâmetro clínico	Captopril		Clonidina		*p*
	**Média ± DP**	**Variação**	**Média ± DP**	**Variação**	
**PAS (mmHg)**					
**PAD (mmHg)**					
**PAM (mmHg)**					
**FC (bpm)**					

PAS: pressão arterial sistólica, PAD: pressão arterial diastólica, PAM: pressão arterial média, FC: frequência cardíaca, mmHg: milímetro de mercúrio, bpm: batimentos por minuto, DP: desvio padrão, p: teste estatístico.

Figura 5. Curva de Sobrevida (análise de Kaplan-Meyer) para evolução dos níveis tensionais em puérperas usando captopril ou clonidina

VII – CRONOGRAMA

MESES<br>ETAPAS	1*	2	3	4	5	6	7	8	9	10	11	12
Elaboração do projeto	X											
Qualificação do projeto		X										
Comitê de Ética			X	X								
Preparaçào da equipe e teste dos formulários			X	X								
Revisão da literatura	X	X	X	X	X	X	X					
Preparo das medicações				X								
Coleta dos dados					X	X	X	X				
Revisão e correção dos formulários preenchidos					X	X	X	X				
Digitação						X	X	X				
Revisão da digitação, limpeza e testes de consistência						X	X	X				
Tabulação e análise dos dados							X	X				
Revisão da análise dos dados								X	X	X	X	
Redação da tese									X	X	X	
Revisão da tese / preparação para publicação										X	X	
Defesa de tese											X	
Envio para publicação												X

* o mês “1” deverá corresponder ao mês de abril do ano de 2012

VIII. ORÇAMENTO

Os comprimidos de captopril e clonidina serão adquiridos em um laboratório farmacêutico que dispensa essas drogas para o IMIP. O projeto será apresentado ao Edital Universal do CNPq para aprovação, caso aconteça, o orçamento deverá ser financiado por este órgão de fomento. Vale a pena salientar que independente da aprovação desse Edital, o estudo será conduzido conforme disposto no cronograma. O estudo não implicará em gastos adicionais para o IMIP, uma vez que todos os procedimentos serão realizados pelos médicos que já exercem suas atividades habituais no CAM-IMIP, durante o período diurno, de segunda a sexta-feira. Não haverá interferência na conduta do serviço. Os gastos com medicações, formulários, impressos etc. serão de inteira responsabilidade do pesquisador, a menos que o projeto consiga obter financiamento de alguma agência em tempo hábil para a conclusão do Doutorado.

Item	Quantidade	Preço	
		Unitário	Total
**1. Material Permanente**			
**1.1 Equipamento e material permanente**			
Tensiômetro Tycos	02	320,00	640,00
Estetoscópio Littman	02	509,00	1.018,00
Monitor multiparâmetro Dixtal	01	4.000,00	4.000,00
Mouse	01	50,00	50,00
Impressora Multifuncional Laserjet	01	1.699,00	1.699,00
Gravador e Reprodutor de DVD - RX38D - c/ Progressive Scan -	01	500,00	500,00
Monitor e Gabinete<br>Alta performance para programas pesados	01	2.000,00	2.000,00

Teclado Comfort Curve 2000	01	100,00	100,00
**1.2 Material Bibliográfico**			
Solicitação de artigos científicos	30	30,00	900,00
**Subtotal material permanente**			**10.907,00**
**2. Custeio**			
**2.1 Serviços de terceiros**			
Entrevistador (por entrevista)	200	20,00	4.000,00
Estatístico	02	2.000,00	4.000,00
Tradutor (por lauda)	20	40,00	800,00
**2.2 Medicamento**			
Captopril	350	0,05	17,50
Clonidina	350	0,05	17,50
Tubo para Proteinúria de fita (Labstix)	04	100,00	400,00
**2.3 Material de consumo**			
Medcalc	01	3.700,00	3.700,00
Caixas pequenas de papelão	200	0,10	20,00
Tubos para armazenamento das medicações	200	0,50	100,00
Papel A4 (Resma)	20	15,00	300,00
Pen drive	01	75,00	75,00
Cartucho preto	10	50,00	500,00
Cartucho colorido	10	40,00	400,00
CD RW	10	2,5	25,00
Canetas	20	0,70	14,00
Corretivo	04	4,00	16,00
Grampeador	02	30,00	60,00
Clips 6 pacote com 20 unidades	10	2,00	20,00
Grampos (caixa)	04	20,00	80,00
Pasta Arquivos/colecionador	10	3,00	30,00
Etiquetas rolos	2	1,50	3,00
Borracha pacote com 2	05	3,00	6,00
Prancheta	02	14,00	28,00
Apontador	04	3,50	14,00
Lápis pacote com 6	04	4,50	18,00
Calculadora	02	35,00	70,00
**Subtotal custeio**			**14.714,00**
**Total (custeio + material permanente)**			**25.621,00**

JUSTIFICATIVA DO ORÇAMENTO

- Monitor, gabinete, teclado, mouse, impressora e gravador/leitor de CD: utilizado para construção do banco de dados, manutenção do programa Medcalc e análise estatística, além do acesso a internet para pesquisa de periódicos e artigos relacionados ao projeto.

- Tensiômetro e Estetoscópio e monitor multiparâmetro: equipamentos necessários para aferição da pressão arterial.
- Solicitação de artigos científicos: necessários para a construção da discussão da pesquisa.
- Material de consumo (papelaria e cartucho): material necessário para impressão dos formulários, de relatórios técnico-científicos, capacitação e atividades preparação da equipe.
- Material de Consumo (medicação captopril e clonidina): fundamental para o desenvolvimento da pesquisa, que será produzido por um laboratório farmacêutico.
- Tudos de armazenamento: necessários para o armazenamento adequado das capsulas.
- Caixas de papelão: necessárias para guardar os tubos de armazenamento contendo as medicações.
- Fita para proteinúria: necessária para identificar a presença de proteínas na urina, fundamental para o diagnóstico de pré-eclâmpsia.
- Programa Medcalc: que será utilizado para a análise estatística.
- Estatístico: responsável por toda a análise estatística do projeto.
- Entrevistadores: necessários para preenchimentos dos formulários. Do início do diagnóstico até o nascimento do recém-nascido.
- Tradutor: necessário para a tradução do artigo final (do português para o inglês) a ser publicado em revista internacional de alto impacto.

IX. REFERÊNCIAS

1. Steyn DW, Steyn P. Low-dose dopamine for women with severe pre-eclampsia. Cochrane Database of Systematic Reviews. In: *The Cochrane Library,* Issue 05, Art. No. CD003515. DOI: 10.1002/14651858.CD003515.pub1.
2. National High Blood Pressure Education Program. Report of the National High Blood Pressure Education Program Working Group on High Blood Pressure in Pregnancy. Am J Obstet Gynecol. 2000; 183:S1-S22.
3. Amorim MMR, Souza ASR, Katz L, Noronha Neto C. Planned caesarean section versus planned vaginal delivery for severe preeclampsia (Protocol for a Cochrane Review). In: *The Cochrane Library,* Issue 05, 2012.
4. Souza ASR, Amorim MR, Costa AAR, Noronha Neto C. Tratamento anti-hipertensivo na gravidez. Acta Med Port. 2010; 23(1):077-084.
5. Nascimento JC, Fernandes F, Sass N. Aspectos imunogenéticos da pré-eclâmpsia. Femina. 2001; 29: 673-5.
6. Romero JP. Disfunción endotelial en la preeclampsia. An Fac Med (Perú). 2003; 64:43-54
7. Solomon CG, Seely EW. Hypertension in pregnancy. Endocrinol Metab Clin N Am. 2006; 35:157-71
8. Batista ECS. Perfil epidemiológico e resultados perinatais em pacientes com síndromes hipertensivas na gravidez [dissertação]. Fortaleza: Departamento de Saúde Materno-Infantil da Universidade Federal do Ceará; 2009.
9. Melo BCP, Amorim MMR, Katz L, Coutinho I, Veríssimo G**.** Perfil epidemiológico e evolução clínica pós-parto na pré-eclâmpsia grave. Rev Assoc Med Brás. 2009; 55(2):175-80.

10. Adusumalli J, Han CS, Beckham S, Bartholomew ML, Williams J. Chorionic villus sampling and risk for hypertensive disorders of pregnancy. Am J Obstet Gynecol**.** 2007; 196:591-7.

11. Larsen WI, Strong JE, Farley JH. Risk factors for late postpartum preeclampsia. J. Reprod. Med. 2012; 57(1-2): 35-8.

12. Lei Q, Lv LJ, Zhang BY, Wen JY, Liu GC, *et. al*. Ante-partum and post-partum markers of metabolic syndrome in pre-eclampsia. J Hum Hypertens. 2011; 25(1): 11-7.

13. The Seventh Report on the Joint National Comitte Prevention Detection Evaluation and Treatment of High Blood Pressure (JNC7). National High Blood Pressure Education Program. [cited 2008 sept]. Avaliable from: http://www.nhlbi.nih.gov/guidelines/hypertension/express.pdf.

14. Martins Jr JN, Thigpen BD, Moore RC, Rose CH, Cushmam J, May AW. Stroke and severe preeclampsia and eclampsia: A paradigm shift focusing on systolic blood pressure. Obstet Gynecol. 2005;105: 246-54.

15. Firoz T, Melnik T. Postpartum evaluation and long term implications. Best Pract Res Clin Obstet Gynaecol. 2011; 25(4): 549-61.

16. Melchiorre K, Sutherland GR, Liberati M, Thilaganathan B. Preeclampsia is associated with persistent postpartum cardiovascular impairment. Hypertension. 2011; 58(4): 709-15.

17. Al-Safi Z, Imudia AN, Filetti LC, Hobson DT, Bahado-Singh RO, *et. al.* Delayed postpartum preeclampsia and eclampsia: demographics, clinical course, and complications. Obstet Gynecol. 2011; 118(5): 1102-7.

18. Podymow T, August P. Postpartum course of gestational hypertension and preeclampsia. Hypertens Pregnancy. 2010; 29(3): 294-300.

19. Oliveira MG, Noblat ACB, Noblat L, Passos LC. Análise da prescrição de captopril em pacientes hospitalizados. Arq. Bras. Cardiol. 2008; 91(6): 238-46

20. Van den Born BJ, Beutler JJ, Gaillard CA, de Gooijer A, van den Meiracker AH. *et. al.* Dutch guideline for the management of hypertensive crisis -- 2010 revision. Neth J Med. 2011; 69(5): 248-55.

21. Save the Children. Sate of the World's Mothers 2007. [citado 2007 may]. Avaliable from: http://www.savethechildren.org/publications/mothers/2007/SOWM-2007-final.pdf.

22. Magee L, Sadeghi S. Prevention and treatment of postpartum hypertension Cochrane Database Syst Rev. 2005;(1):CD004351.

23. Vigil-De Gracia P, Ruiz E, LA pez JC, de Jaramillo IA, Vega-Maleck JC, *et. al.* Management of severe hypertension in the postpartum period with intravenous hydralazine or labetalol: a randomized clinical trial. Hypertension in pregnancy. 2007; 26(2): 163-71.

24. Pires JGP, Almeida PJ. Drogas anti-hipertensivas e gravidez. J. bras. ginecol. 1996; 96(10): 493-6

25. Easterling TR, Carr DB, Davis C, Diederichs C, Brateng DA, *et. al.* Low-dose, short-acting, angiotensin-converting enzyme inhibitors as rescue therapy in pregnancy. Obstet Gynecol. 2000; 96(6): 956-61.

26. Germany PL, Martins SC. Tratamento anti-hipertensivo na gestação e lactação. Femina. 2003; 31(9): 803-8.

27. Santello JL, Mion DJr. Captopril Associado à Hidroclorotiazida no Tratamento da Hipertensão Leve e Moderada. Estudo Multicêntrico Brasileiro. Arq. Bras. Cardiol. 1998; 71(5): 713-6

28. Jordão MG, Santos ATL. Hipotensão arterial em cirurgia de revascularização do miocárdio: influência dos inibidores da enzima conversora de angiotensina. Rev. Bras. Anestesiol. 2002; 52(2): 209-16

29. Canchola O, Hugo V, Heredia R, Lourdes M, de la Vega C. Comparación de la eficacia entre captopril sublingual contra placebo en urgencias hipertensivas. Med. interna Méx. 2000; 16(6): 303-7.

30. Vanegas L, José B. Eficacia del captopril sublingual en comparación con el captopril por vía oral en el control de la urgencia hipertensiva en los pacientes ingresados en la emergencia de medicina interna del HEODRA, en el período de agosto del 2003 a enero del 2004. León; s.n; 2004. 35 p. tab. Monografia [LILACS ID: 383075]

31. Moritz RD, Queiroz LP, Pereira MR, Scotinni MA. Estudo comparativo do uso da nifedipina e do captopril em urgências hipertensivas. Arq Bras Cardiol. 1989; 52(6): 323-6.

32. Vásquez D, Quijano F, Durán M. Uso del captopril en la crisis hipertensiva inducida por el embarazo. Rev Colomb Obstet Ginecol. 1995; 46(1): 41-7.

33. Barrilleaux PS, Martin Jr JN. Hypertension therapy during pregnancy. Clinical Obstet Gynecol. 2002; 45: 22-34.

34. Marui S, Oliveira CHMC, Souza SCAL, Berger K, Khawal C, *et. al.* Tolerância ao teste da clonidina em 180 pacientes: estudo da eficácia da expansão volêmica para o controle de hipotensão arterial. Arq Bras Endocrinol Metab. 2005; 49(4): 510-5

35. Benchimol CB, Tabet AL. Comparação entre maleato de enalapril e cloridrato de clonidina no tratamento da hipertensäo leve a moderada/ Comparison in to

enalapril maleate and clonidin hydrochlorine in the treatment of light to moderate hypertension. Rev Bras Med. 1987; 44(6): 166-8.

36. Tuimala R, Punnonen R, Kauppila E. Clonidine in the treatment of hypertension during pregnancy. Ann Chir Gynaecol Suppl.1985; 197: 47-50.
37. McMahon FG, Jain AK, Vargas R, Fillingim J. A double-blind comparison of transdermal clonidine and oral captopril in essential hypertension. Clinical therapeutics. 1990; 12(2): 88-100.
38. Simoni RF, Cangiani LM, Pereira AMSA, Abreu MP, Cangiani LHC, *et. al*. Eficácia do emprego da metadona ou da clonidina no intraoperatório para controle da dor pós-operatória imediata após uso de remifentanil Rev. Bras. Anestesiol. 2009; 59(4):
39. Cruz JRS, Cruz DFBM, Branco BC, Santiago AEQ, Amaral JLG. Clonidina como medicação pré-anestésica em facectomias: comparação entre as doses de 100 µg e 200 µg. Rev. Bras. Anestesiol. 2009; 59(6): 694-703
40. Rocha APC, Barros GAM, Nascimento JS, Santos KP, Vasconcelos LM, *et. al*. Sedação com sufentanil e clonidina em pacientes submetidos a cateterismo cardíaco. Arq. Bras. Cardiol. 2011; 96(3):
41. Stocche RM, Garcia LV, Klamt JG, Pachione A, Yu HH, *et. al.* Comparação entre nifedipina por via sublingual e clonidina por via venosa no controle de hipertensão arterial peri-operatória em cirurgias de catarata Rev. Bras. Anestesiol. 2002; 52(4): 426-33
42. Santos LC, Porto AMF, Amorim MM, Guimarães V. Obstetrícia Diagnóstico e Tratamento - Instituto Materno Infantil de Pernambuco (IMIP). Rio de Janeiro: MEDSI; 1998.

43. Amorim MMR, Katz L, Ávila MB, Araújo DE, Valença M, Albuquerque CJM, Carvalho ARMR, Souza ASR. Perfil das admissões em uma unidade de terapia intensiva obstétrica de uma maternidade brasileira. Rev Bras Saúde Mater Infant. 2006; 6: 55S-62S.
44. Atalah E, Castillo CL, Castro RS, Amparo Aldea P. Propuesta de un Nuevo estándar de evaluación nutritional de embarazadas. Rev Med Chile. 1997; 125:1429-36.
45. Ministério da Saúde. Pré-Natal e Puerpério: atenção qualificada e humanizada - manual técnico. Brasília: Ministério da Saúde; 2005. p. 40-8.
46. Dicionário de especialidades farmacêuticas: DEF 2006/7-35 ed. Rio de Janeiro: Ed. de Publicações Biomédicas; 2007.
47. Moher D, Schultz KF, Altman DG. The CONSORT statement: revised recommendations for improving the quality of reports of parallel-group randomized trials. Lancet 2001; 357:1191-4.
48. Zuspan FP. Problems encountered in the treatment of pregnancy-induced-hypertension. A point of view. Am J Obstet Gynecol 1978; 131:591-97.
49. Santos LC, Amorim MM, Katz L, Albuquerque CJ. Pré-eclâmpsia e Eclâmpsia. In: Santos LC, Amorim MM, Katz L, Albuquerque CJ, editores. Terapia Intensiva em Obstetrícia - Instituto Materno Infantil de Pernambuco (IMIP). Rio de Janeiro: Medsi; 2004. p. 37-61.
50. World Medical Association: Declaration of Helsinki. JAMA 1997; 277:925-6.
51. Brasil. Ministério Nacional da Saúde. Conselho Nacional de Saúde. Resolução 196/96 sobre pesquisa envolvendo seres humanos. Bioética 1996; 4:S15-S25.

APÊNDICE 1

AVISO AOS OBSTETRAS DO CAM-IMIP

Diante do projeto de pesquisa intitulado "**EFETIVIDADE DA CLONIDINA PARA TRATAMENTO HIPOTENSOR DE EMERGÊNCIA NO PUERPÉRIO: ENSAIO CLÍNICO RANDOMIZADO**" do aluno *Carlos Noronha Neto*, matriculado na turma 2009-2013 do doutorado em Saúde Materno Infantil do Instituto de Medicina Integral Prof. Fernando Figueira:

Vimos por meio deste, informar que todas as puérperas, com síndromes hipertensivas da gestação apresentando picos hipertensivos, internadas na UTI obstétrica do IMIP deverão ser comunicadas ao responsável pela pesquisa, Carlos Noronha Neto, através do telefone 9234-0457. Comunicamos ainda, que devido à pesquisa, manteremos como agente hipotensor para pico hipertensivo o captopril ou a clonidina.

Certos da compreensão e colaboração de todos, agradecemos antecipadamente.

Recife, 01 de agosto de 2012.

____
Vilma Guimarães
Coordenadora do Serviço de Tocoginecologia do IMIP

____
Carlos Noronha Neto
Pesquisador

APÊNDICE 2

LISTA DE CHECAGEM

"EFETIVIDADE DA CLONIDINA PARA TRATAMENTO HIPOTENSOR DE EMERGÊNCIA NO PUERPÉRIO: ENSAIO CLÍNICO RANDOMIZADO"

Puérpera nº. ☐☐☐
Formulário nº. ☐☐☐
Pesquisador: Carlos Noronha Neto
Data: ☐☐/☐☐/☐☐☐☐

IDENTIFICAÇÃO

Nome: ____ Registro: ☐☐☐☐☐☐☐☐

Idade: ☐☐ anos

CRITÉRIOS DE INCLUSÃO

Síndrome hipertensiva da gestação	☐ **SIM**	☐ **NÃO**
Pressão arterial instável	☐ **SIM**	☐ **NÃO**
Puérpera	☐ **SIM**	☐ **NÃO**

CRITÉRIOS DE EXCLUSÃO

Doenças maternas associadas	☐ **SIM**	☐ **NÃO**
Qual ? ____		
Tabagismo	☐ **SIM**	☐ **NÃO**
Uso de outras medicações hipotensoras ou drogas ilícitas que possam interferir na hemodinâmica materna.	☐ **SIM**	☐ **NÃO**
Qual ? ____________		
Contra-indicações ao uso da clonidina.	☐ **SIM**	☐ **NÃO**
Qual ? ____		
Contra-indicações ao uso da captopril.	☐ **SIM**	☐ **NÃO**
Qual ? ____		

APTA A PARTICIPAR DO ESTUDO?

☐ **SIM** ☐ **NÃO**

APÊNDICE 3

TERMO DE CONSENTIMENTO LIVRE E ESCLARESCIDO

(De acordo com os critérios da resolução 196/96 do Conselho Nacional de Saúde)

Cara senhora, você está sendo convidada como voluntária a participar da pesquisa:

"EFETIVIDADE DA CLONIDINA PARA TRATAMENTO HIPOTENSOR DE EMERGÊNCIA NO PUERPÉRIO: ENSAIO CLÍNICO RANDOMIZADO"

A JUSTIFICATIVA, OS OBJETIVOS E OS PROCEDIMENTOS

O motivo que nos leva a estudar o problema é a grande quantidade de mulheres no resguardo que se sentem mal com a crise de pressão arterial alta. O tratamento da crise de pressão alta durante o resguardo é realizado com um remédio chamado captopril que funciona de forma ideal para baixar a pressão, porém aqui na UTI obstétrica outro remédio, que também é empregado em pessoas que têm pressão alta, está sendo feito e seus resultados estão surpreendendo a equipe de médicos. Não existem estudos comprovando que o tratamento da crise de pressão alta no resguardo é melhor com o captopril ou com a clonidina e ainda não se sabe se os efeitos colaterais da clonidina são piores que os do captopril. Desta forma, o objetivo deste trabalho é estudar o efeito do tratamento na crise de pressão alta nas mulheres no resguardo com clonidina, comparando com o captopril.

A coleta de dados será da seguinte forma: as mulheres no resguardo, que passam mal com a crise de pressão alta, serão sorteadas em dois grupos; um grupo irá fazer o tratamento com o captopril e o outro grupo com a clonidina. Assim, participando do estudo, poderei estar sendo tratada com a medicação captopril ou com a clonidina, independente da minha vontade. Diariamente todas as mulheres no resguardo serão

examinadas e exames complementares serão solicitados segundo o protocolo do trabalho.

DESCONFORTOS, RISCOS E BENEFÍCIOS

Como já se sabe do benefício do tratamento da crise de pressão alta durante o resguardo com o captopril, os benefícios com a clonidina ainda não foi bem determinado, por isso esse trabalho é de grande importância. A crise de pressão alta durante o resguardo pode levar a mulher a ter derrame, infarto e pior do que isso a morte. Entretanto, esse tratamento pode ser feito com os dois remédios sem maiores problemas. O tratamento das crises de pressão alta será realizado nos dois grupos diminuindo os riscos dessas complicações descritas acima. Mulheres no resguardo já com as complicações da crise de pressão alta diagnosticadas no início do estudo não estáveis não participarão desse trabalho.

A captopril poderá ocasionar possíveis efeitos colaterais leves sem comprometer o bem estar da mulher no resguardo e é o remédio atualmente feito no IMIP para tratamento da crise de pressão alta e a clonidina pode também provocar alguns efeitos claterais que precisam ser mais bem estudados. Os exames laboratoriais já são realizados de forma rotineira no IMIP e a mulher não será exposta a colheitas de sangue solicitadas de forma desnecessárias.

FORMA DE ACOMPANHAMENTO E ASSISTÊNCIA

A senhora será acompanhada por uma equipe formada por obstetras, com experiência em terapia intensiva, clínicos, cardiologistas e quando necessário nefrologista e neurologista, todos especialistas.

Durante a realização da pesquisa (acompanhamento no resguardo) não haverá alteração da conduta habitualmente utilizada no IMIP, apenas será acrescido o captopril ou clonidina, como medicação para o tratamento da crise de pressão alta.

GARANTIA DE ESCLARECIMENTO, LIBERDADE DE RECUSA E GARANTIA DE SIGILO

A senhora será esclarecida sobre a pesquisa e sobre o que desejar. A senhora é livre para se recusar em participar, retirar seu consentimento ou interromper a participação a qualquer momento. A sua participação é voluntária e a recusa não irá acarretar qualquer penalidade ou perda de benefícios.

O(s) pesquisador(es) irá(ão) tratar a sua identidade com padrões profissionais de sigilo. Seu nome ou o material que indique a sua participação não será liberado sem a sua permissão. Você não será identificada em nenhuma publicação que possa resultar deste estudo. Uma cópia deste consentimento informado será arquivada no seu prontuário.

CUSTOS DA PARTICIPAÇÃO, RESSARCIMENTO E INDENIZAÇÃO POR EVENTUAIS DANOS

A participação no estudo não acarretará custos para a senhora. Todos os exames serão realizados pelo IMIP e a senhora poderá receber ressarcimento ou indenização, segundo as normas legais, para qualquer situação em que se sinta lesada.

DECLARAÇÃO DA PARTICIPANTE

Eu, ____ puérpera matriculada no IMIP com o registro □□□□□□□□, declaro que fui informada dos objetivos e finalidade da pesquisa "**EFETIVIDADE DA CLONIDINA PARA**

TRATAMENTO HIPOTENSOR DE EMERGÊNCIA NO PUERPÉRIO: ENSAIO CLÍNICO RANDOMIZADO" maneira clara e detalhada e esclareci minhas dúvidas. Sei que em qualquer momento poderei solicitar novas informações e alterar a minha decisão, sem que isso venha a prejudicar meu atendimento no IMIP. O(a) pesquisador(a) ____ certificou-me de que todos os dados desta pesquisa serão confidenciais. Também sei que caso existam gastos adicionais, estes serão absorvidos pelo pesquisador responsável. Em caso de dúvidas poderei chamar o estudante ____________________________________ ou o pesquisador Dr. Carlos Noronha Neto no telefone (81) 9234-0457 ou contactar o Comitê de Ética em Pesquisa do IMIP, número 2122-4702. Declaro ainda que concordei em seguir todas as orientações do pesquisador, a realização da medicação e dos exames laboratoriais nos prazos previstos, que concordei em participar desse estudo, que recebi uma cópia deste termo de consentimento livre e esclarecido e me foi dada a oportunidade de ler e esclarecer as minhas dúvidas.

____

Nome Assinatura do Participante Data

____

Nome Assinatura do Pesquisador Data

____

Nome Assinatura da Testemunha Data

APÊNDICE 4

FORMULÁRIO DE PESQUISA

"EFETIVIDADE DA CLONIDINA PARA TRATAMENTO HIPOTENSOR DE EMERGÊNCIA NO PUERPÉRIO: ENSAIO CLÍNICO RANDOMIZADO"

DADOS DA PESQUISA

Formulário nº. □□□

Pesquisador: Carlos Noronha Neto

Número da caixa: □□□

IDENTIFICAÇÃO

Nome: ____

Registro: □□□□□□□□

Local: Instituto de Medicina Integral Prof. Fernando Figueira.

Data de Nascimento: □□/□□/□□□□

Endereço: ____

Telefone: (□□)□□□□-□□□□

Data de admissão: □□/□□/□□□□

DADOS GERAIS

Idade: □□ anos

Gesta: □□ Para: □□ Aborto: □□

DADOS DO DIAGNÓSTICO DE SÍNDROME HIPERTENSIVA DA GESTAÇÃO

Dados gerais da gestação

Idade gestacional do diagnóstico: ☐☐ sem ☐ dias

Tipo da Síndrome hipertensiva: 1. ☐ Pré-eclâmpsia grave

2. ☐ Iminência de eclâmpsia

3. ☐ Hipertensão crônica agravada pela gestação

Idade gestacional da interrupção da gravidez: ☐☐ sem ☐ dias

Variáveis hemodinâmicas maternas na admissão em UTI obstétrica

Frequência cardíaca: 1. ☐☐☐

Pressão arterial sistólica: 1. ☐☐☐

Pressão arterial diastólica: 1. ☐☐☐

Pressão arterial medial: 1. ☐☐☐

Exames laboratoriais na admissão em UTI obstétrica

**Exames**	**Resultados**
Plaquetas / mm^3	
Uréia (mg/dl)	
Creatinina (mg/dl)	
Ácido úrico (mg/dl)	
DHL (U/l)	
AST (U/l)	
ALT (U/l)	

Bilirrubina total (mg/dl)	
Bilirrubina direta (mg/dl)	
Bilirrubina indireta (mg/dl)	
Proteinúria de fita (labstix)	
Sódio	
Potássio	
Cloro	

DADOS DA EVOLUÇÃO/ACOMPANHAMENTO NO PUERPÉRIO

Parâmetros clínicos durante o internamento em UTI obstétrica do IMIP

**Variável**	**Dt**	**Dt**	**Dt**	**Dt**	**Dt**	**Dt**	**Dt**	**Dt**	**Dt**	**Dt**	**Dt**	**Dt**	**Dt**	**Dt**	**Dt**	**Dt**	**Dt**	**Dt**
	**H**	**H**	**H**	**H**	**H**	**H**	**H**	**H**	**H**	**H**	**H**	**H**	**H**	**H**	**H**	**H**	**H**	**H**
FC																		
PAS																		
PAD																		
PAM																		

**Variável**	**Dt**	**Dt**	**Dt**	**Dt**	**Dt**	**Dt**	**Dt**	**Dt**	**Dt**	**Dt**	**Dt**	**Dt**	**Dt**	**Dt**	**Dt**	**Dt**	**Dt**	**Dt**
	**H**	**H**	**H**	**H**	**H**	**H**	**H**	**H**	**H**	**H**	**H**	**H**	**H**	**H**	**H**	**H**	**H**	**H**
FC																		
PAS																		

PAD																		
PAM																		

Exames Laboratoriais durante o internamento em UTI obstétrica do IMIP

**Exames**	**Dt**	**Dt**	**Dt**	**Dt**	**Dt**	**Dt**	**Dt**	**Dt**	**Dt**	**Dt**	**Dt**	**Dt**	**Dt**	**Dt**
	**H**	**H**	**H**	**H**	**H**	**H**	**H**	**H**	**H**	**H**	**H**	**H**	**H**	**H**
Plaquetas / mm^3														
Uréia (mg/dl)														
Creatinina (mg/dl)														
Ac. Úrico (mg/dl)														
DHL (U/l)														
AST (U/l)														
ALT (U/l)														
BT (mg/dl)														
BD (mg/dl)														
BI (mg/dl)														
Proteinúria (labstix)														
Sódio														
Potássio														
Cloro														

Desfechos puerperais

Dias de internamento hospitalar durante o puerpério: ☐☐☐ dias

Dias de utilização do hipotensor: ☐☐☐ dias

Frequência de pressão arterial instável durante o puerpério: ☐☐☐

**Variáveis**	**SIM**	**NÃO**
Edema agudo de pulmão		
Eclâmpsia		
Síndrome HELLP		
Oligúria		
Iminência de eclampsia		
Óbito materno		
Complicações maternas relacionadas às síndromes hipertensivas da gravidez;		
Complicações maternas não relacionadas às síndromes hipertensivas da gravidez		
Coagulação intravascular disseminada		

<u>EFEITOS ADVERSOS DO CAPTOPRIL</u>

**Variáveis**	**SIM**	**NÃO**
Tosse seca;		
Erupções cutâneas;		
Pruridos;		
Febre;		
Artralgia;		

Astenia;		
Fadiga;		
Náusea;		
Tontura;		
Anorexia;		
Dores abdominais;		
Irritação gástrica;		
Insônia;		
Dispnéia;		
Alopecia.		
Distúrbios cardiovasculares (hipotensão, taquicardia, dor torácica);		
Distúrbios hepatobiliares (icterícia, hepatite);		
Distúrbios musculoesqueléticos (mialgia, miastenia);		
Distúrbios dermatológicos (pênfigo bolhoso, eritema multiforme, dermatite esfoliativa).		

EFEITOS ADVERSOS DA CLONIDINA

**Variáveis**	**SIM**	**NÃO**
Secura da boca;		
Sensação de cansaço;		
Distúrbio ortostático;		
Vertigem;		
Obstipação;		
Transtornos do sono;		

Distúrbios circulatórios;		
Transtornos da percepção;		
Pesadelo;		
Ginecomastia;		
Dores das glândulas parótidas;		
Estado de depressão;		
Secura da mucosa nasal;		
Diminuição da lacrimação.		

Data da alta ______/______/_______ Hora: ☐☐:☐☐

Revisado em _____/______/_______

Digitado em _____/______/_______
